# Cardiomyocyte USP20 alleviates septic cardiomyopathy by deubiquitinating and inhibiting NLRP3 activity

**DOI:** 10.1002/ctm2.70494

**Published:** 2025-10-03

**Authors:** Shanshan Dai, Yucong Zhang, Ziyi Huang, Yunxuan Chen, Zexin Yang, Ruihan Zheng, Keke Ye, Lingfeng Zhong, Xiangtao Zheng, Xueli Cai, Weijian Huang

**Affiliations:** ^1^ Department of Cardiology The First Affiliated Hospital of Wenzhou Medical University, The Key Laboratory of Cardiovascular Disease of Wenzhou Wenzhou Zhejiang People's Republic of China; ^2^ Department of Emergency, The Key Laboratory of Emergency and Disaster Medicine of Wenzhou The First Affiliated Hospital of Wenzhou Medical University Wenzhou Zhejiang People's Republic of China; ^3^ Department of Vascular Surgery The Second Affiliated Hospital of Wenzhou Medical University Wenzhou Zhejiang People's Republic of China

**Keywords:** NLRP3, pyroptosis, septic cardiomyopathy, ubiquitin‐specific peptidase 20

## Abstract

**Objectives:**

Although extensive research on septic cardiomyopathy has been conducted, effective therapies are still limited. Ubiquitin‐specific peptidase 20 (USP20), a deubiquitinating enzyme, is critical in regulating protein ubiquitination and various cellular processes. whether USP20 is involved in the pathogenesis of septic cardiomyopathy remains unclear. This study investigated the impact of USP20 on septic cardiomyopathy.

**Methods:**

The cardiomyocyte‐specific USP20 knockout mice (USP20CKO) and NLRP3 knockout mice (NLRP3‐/‐) were used in the present study. A sepsis mouse model was established using lipopolysaccharide (LPS) administration and the cecal ligation and puncture (CLP) procedure. Recombinant adeno‐associated virus serotype 9 (AAV9) was used to achieve overexpression of USP20. Myocardial function, histopathological changes, and pyroptosis levels in heart tissues were evaluated. Liquid chromatography tandem mass spectrometry (LC‐MS/MS) analysis and co‐immunoprecipitation (co‐IP) were performed to identify the molecular mechanism of USP20 in septic cardiomyopathy.

**Results:**

Our results showed that USP20 was downregulated in the myocardium of septic mice. Cardiomyocyte‐specific USP20 deficiency worsened myocardial injury and cardiac dysfunction induced by LPS and CLP. LC‐MS/MS analysis and co‐IP revealed NLRP3 as a substrate protein of USP20. Mechanistically, USP20 removed K63‐linked ubiquitin from K243 via its active site C154, inhibiting NLRP3's interaction with ASC and suppressing its activation and subsequent pyroptosis. Moreover, overexpressing USP20 in cardiomyocytes reduced LPS‐induced myocardial injury. Additionally, the protective effect of USP20 against LPS‐induced damage was nullified in the absence of NLRP3 in mice.

**Conclusions:**

These findings suggest that cardiomyocyte‐derived USP20 is crucial in septic cardiomyopathy progression and may serve as a novel therapeutic target for managing septic cardiomyopathy.

**Key points:**

- Cardiomyocyte‐derived USP20 is crucial in septic cardiomyopathy progression.
- NLRP3 is identified as a substrate protein of USP20.
- USP20 deubiquitinates NLRP3 by removing K63‐linked ubiquitin at K243 residue via its active site C154, disrupting the interaction between NLRP3 and ASC, suppressing NLRP3 activation and subsequent pyroptosis.
- USP20 may serve as a novel therapeutic target for managing septic cardiomyopathy

## Graphical abstract

Our findings indicated that USP20 was downregulated in the myocardial tissues of septic mice. Cardiomyocyte‐specific deficiency of USP20 exacerbated myocardial injury and cardiac dysfunction induced by LPS and CLP. Conversely, the overexpression of USP20 in cardiomyocytes alleviated LPS‐induced myocardial damage. Furthermore, USP20 was identified as a significant regulator of pyroptosis triggered by LPS. Mechanistically, USP20 deubiquitinated NLRP3 by removing K63‐linked ubiquitin at the K243 residue through its active site C154. This action inhibited the interaction between NLRP3 and ASC, consequently suppressing NLRP3 activation and subsequent pyroptosis. These findings clearly demonstrate that USP20 plays a critical role in the pathogenesis of sepsis‐induced myocardial injury. A schematic diagram illustrating these principal findings is presented in the Graphic Abstract.

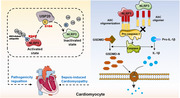

## 1 INTRODUCTION

Septic cardiomyopathy is a prevalent complication of sepsis, significantly impacting patient prognosis.^1^ Patients diagnosed with septic cardiomyopathy exhibit left ventricular systolic dysfunction.^2^ The reported incidence of septic cardiomyopathy among individuals suffering from sepsis ranges between 20% and 60%, with an elevated mortality rate observed in those who experience septic myocardial injury compared to their counterparts without such injury.^3^, ^4^, ^5^ The pathogenesis underlying myocardial dysfunction in the sepsis has yet to be fully elucidated. Despite extensive clinical and basic research conducted on this issue, the therapeutic outcomes for septic cardiomyopathy remain considerably limited, likely due to its intricate mechanisms. Therefore, it is crucial to investigate the pathophysiological processes responsible for septic cardiomyopathy and to explore effective therapeutic strategies.

Protein ubiquitination represents one of the most significant post‐translational modifications, playing a crucial role in regulating protein degradation and activity while contributing to the maintenance of normal physiological processes.^6^, ^7^ Deubiquitylation is the reverse process of ubiquitination, which is catalysed by deubiquitinating enzymes (DUBs) to remove the ubiquitin moieties from substrate proteins^8^ The equilibrium between ubiquitination and deubiquitination is essential for maintaining protein homeostasis and plays a critical role in the regulation of various diseases.^9^, ^10^ Recently, a growing body of research has demonstrated that DUBs play crucial roles in the pathogenesis of sepsis.^11^, ^12^, ^13^, ^14^ Notably, it has been reported that ubiquitin‐specific protease 7 (USP7) enhances NLRP3 expression, thereby exacerbating myocardial injury related to sepsis.^15^ Research indicates that DUBs are involved in various pathophysiological processes leading to septic cardiomyopathy.^16^ Dysregulation of DUBs activity disrupts the homeostasis of effector protein, potentially resulting in myocardial injury. Recent studies have shown that specific DUBs mediate the deubiquitination of key proteins in the inflammatory signalling pathways of sepsis, thereby regulating the inflammatory response^17^, ^18^, ^19^ Furthermore, DUBs have been identified to regulate the stability or activity of relevant proteins, thereby influencing mitochondrial function and cell death.^12^, ^15^, ^20^, ^21^ The above processes are associated with the onset and progression of septic cardiomyopathy. Deubiquitination may emerge as a significant factor contributing to myocardial injury in the context of septic conditions. Consequently, gaining insight into the role of DUBs in septic cardiomyopathy may offer valuable therapeutic targets for the future management of myocardial injury in patients suffering from sepsis.

In this study, we observed a reduced expression of ubiquitin‐specific protease 20 (USP20) in cases of septic cardiomyopathy. This finding suggests that USP20 may be involved in myocardial injury associated with sepsis. USP20 is a crucial member of the ubiquitin‐specific protease family, which plays a significant role in regulating protein ubiquitination and thus modulates various cellular processes such as cell survival, proliferation and inflammation.^22^ The physiological functions of USP20 have been identified in several conditions, including neurological diseases, metabolic disorders and cancer.^23^, ^24^, ^25^, ^26^ USP20 mitigates tumour necrosis factor (TNF)‐induced inflammation in smooth muscle cells and reduces the progression of atherosclerosis.^27^ In addition, USP20 contributes to maintain endoplasmic reticulum (ER) homeostasis by deubiquitinating and stabilizing reticulophagy regulator 1 (RETREG1).^28^ However, the involvement of USP20 in the pathogenesis of septic cardiomyopathy remains unreported.

In the present study, we aim to investigate the role of USP20 in sepsis‐induced cardiomyopathy and explore the underlying mechanisms. Our findings may contribute to the development of novel therapeutic strategies for managing septic cardiomyopathy.

## 2 MATERIALS AND METHODS

### 2.1 Animal experiments

The cardiomyocyte‐specific USP20 knockout mice (USP20CKO) was generated by Gem Pharmatech Co., Ltd. The genotype of USP20CKO was maintained by crossing the C57BL/6JGpt‐*Usp20^em1Cflox^*/Gpt mouse (USP20^fl/fl^, strain No. T052123) and the C57BL/6JGpt‐*H11^em1Cin (Myh6‐iCre)^*/Gpt mouse (Myh6‐Cre, strain No. T004713). The C57BL/6JGpt‐*Nlrp3^em7Cd4411^*/Gpt mice (NLRP3^−/−^, strain NO. T010873) and their wild‐type (WT) littermates (8‐week‐old and male) were purchased from Gem Pharmatech Co., Ltd. Mice were maintained in a specific pathogen‐free (SPF) environment with controlled temperature (25°C) and had unrestricted access to water and food. All animal experiments were conducted and analysed by an investigator who was blinded to the group assignments. Mice were assigned to groups using randomization techniques.

After acclimatization to the environment, mice were administered 10 mg/kg lipopolysaccharide (LPS, L2630‐25 mg, Escherichia coli O111:B4, Merck KGaA) via intraperitoneal injection. In contrast, the control group received an equivalent volume of sterile saline through the same route of administration. Additionally, a sepsis mouse model was established using the cecal ligation and puncture (CLP) method to further elucidate the role of USP20. Briefly, following anaesthesia, the mice were positioned supine on an operating board. The hair at the surgical site was shaved, and the area was disinfected with 75% alcohol three times. A longitudinal incision measuring 0.6–1.0 cm was made along the linea alba in the abdominal wall using ophthalmic scissors. Both skin and fascia were incised to reveal the abdominal cavity. Subsequently, the cecum was freed from surrounding tissue and ligated approximately 1 cm distal to its end. A 21‐gauge needle was employed to puncture into the cecum's tip. Thereafter, bowel contents were returned to the abdominal cavity before suturing closed both fascia and skin layers. The sham group underwent laparotomy for isolation of the cecum without any ligation or puncture procedures performed. 24 h after LPS or CLP treatment, cardiac function in surviving mice was assessed prior to sacrifice. Serum and myocardial tissues were subsequently harvested for further experimentation.

To achieve specific overexpression of USP20 in mouse heart tissue, we used a recombinant adeno‐associated virus serotype 9 (AAV9) containing a cardiac‐specific promoter, cardiac troponin T (cTNT, designated as AAV9‐cTNTp‐MCS‐3Flag‐T2A‐EGFP; GV571, Genechem Co., Ltd.), alongside a plasmid that encodes USP20 (referred to as AAV9‐cTnT‐USP20^oe^) or an empty vector control (EV). The mice were subjected to tail vein injection with AAV9‐cTnT‐USP20^oe^ at a dosage of 1 × 10^11^ v.g./month, one month prior to the LPS treatment.

### 2.2 Echocardiography

Mice were anaesthetized using 3% isoflurane and positioned on a warming platform for echocardiographic assessment. Ventricular motion was recorded via M‐mode echocardiography in a standard short‐axis view employing a multimodal small animal ultrasound imaging system (Vevo 3100, FUJIFILM VisualSonics). Ejection fraction (EF) and fractional shortening (FS) were subsequently measured.

### 2.3 Serum biochemical analysis

The serum levels of cardiac troponin I (cTnI, E‐EL‐M1801, Elabscience), creatine kinase isoenzyme MB (CK‐MB, E006‐1‐1, Jiancheng Biological Engineering Institute), lactate dehydrogenase (LDH, BC0685, Solarbio), and interleukin‐1β (IL‐1β, F10770) were evaluated using commercial kits in accordance with the manufacturer's instructions.

### 2.4 Cell experiments

Isolation of neonatal rat cardiomyocytes (NRCMs) was achieved by dissecting the left ventricle of neonatal Sprague–Dawley (SD) rats, following established protocols.^29^ NRCMs, NIH/3T3 (GNM 6, Shanghai Institute of Biochemistry and Cell Biology), and HL‐1 cell lines (GNM46, Shanghai Institute of Biochemistry and Cell Biology) were incubated in DMEM (C11995500BT, GIBCO) supplemented with 10% fetal bovine serum (FBS, F2442, Sigma‐Aldrich), 100 U/mL penicillin, and 100 U/mL streptomycin (15140122, Thermo Fisher) at 37°C in a 5% CO_2_ atmosphere.

Upon reaching the beating stage, the cells were stimulated with LPS at a concentration of 10 µg/mL for 6 hours, followed by treatment with nigericin (Nig, N102401, Aladdin) at a concentration of 10 µM for 1 hour. NRCMs in the control group received an equivalent volume of corresponding solvents for incubation.

To induce the overexpression of USP20, cells were transfected with either the USP20 plasmid (Gene Pharma) or a control vector using Lipofectamine 3000 (cat. no. L3000‐015, Thermo Fisher Scientific) according to the manufacturer's technical specifications. To silence USP20 in cells, small interfering RNA (siRNA) was employed. A total of 50 nM siRNA was transfected in Opti‐MEM™ Medium (cat. no. 31985070, Thermo Fisher Scientific), supplemented with 2 µL Lipofectamine 3000. Following transfection, cells were incubated for a duration of 24 h.

### 2.5 Cell viability assessment

The commercial kits used in this study included the Cell Counting Kit‐8 (CCK‐8, C0037) and the LDH Cytotoxicity Assay Kit (C0016), both sourced from Beyotime. These kits were employed to evaluate cellular viability in accordance with the manufacturer's instructions.

### 2.6 Western blot

Protein samples were extracted from cardiomyocytes and myocardial tissue. A BCA Protein Assay Kit (BP012, Solarbio) was employed to quantify the concentration of proteins. Equal amounts of protein were loaded and separated by sodium dodecyl sulphate‐polyacrylamide gel electrophoresis (SDS‐PAGE). Subsequently, the proteins on the gel were transferred to a polyvinylidene fluoride (PVDF) membrane, which was then incubated in a 5% fat‐free milk solution for 1 h at room temperature for blocking purposes. The membranes were treated with primary antibodies against USP20 (1:1000, ab219800, Abcam), NLRP3 (1:1000, ab263899, Abcam), Caspase‐1 (1:1000, sc‐56036, Santa Cruz Biotechnology), GSDMD (1:1000, ab219800, Abcam), and GAPDH (1:1000, 5174, Cell Signaling Technology) overnight at 4°C. Following this incubation, the membranes were exposed to corresponding secondary antibodies for 1 h at room temperature before being washed three times with TBST. Finally, an enhanced chemiluminescence (ECL) reagent was applied to the membrane surface, and signals were detected using an ECL detection system.

### 2.7 Hematoxylin and eosin (H&E) staining

Heart tissues were fixed in 4% paraformaldehyde at room temperature for 24 h, followed by embedding in paraffin and sectioning into slices with a thickness of 5 µm. After undergoing deparaffinization and rehydration, the slices were subjected to H&E staining using reagents from Solarbio (G1120), following the manufacturer's technical specifications. The samples were subsequently visualized using a microscope.

### 2.8 Immunohistochemical (IHC) staining

Heart tissue sections were processed according to the same protocol used for H&E staining. Following deparaffinization and rehydration, the slices were incubated in citrate buffer (0.01 M, pH 6.0) using a pressure cooker for 10 min after boiling water, and then cooled to room temperature for antigen retrieval. Subsequently, the sections were treated with 3% hydrogen peroxide for 10 min and blocked with 5% bovine serum albumin (BSA) (A8020, Solarbio) at room temperature for 30 min. Thereafter, an overnight incubation with USP20 antibody (dilution of 1:100) was conducted at 4°C, followed by application of the corresponding secondary antibody at room temperature for 1 h. Finally, the slices were subjected to treatment with diaminobenzidine and images were captured using a microscope.

### 2.9 Immunofluorescence (IF) staining

Heart tissue sections were first blocked with 10% FBS and then permeabilized using 0.1% Triton X‐100. Subsequently, the slices were subjected to immunofluorescent staining with USP20 (1:100, A301‐189A‐M, Bethyl), A‐actin (1:100, 48938s, Cell Signaling Technology), Vimentin (1:100, EM0401, HuaBio), and F4/80 (1:200, sc‐377009, Santa Cruz Biotechnology) antibodies overnight at 4°C. Following this incubation period, they were treated with a secondary antibody for 1 h at room temperature. Finally, the sections were preserved with a mounting solution containing DAPI and examined under a confocal laser scanning microscope.

### 2.10 TdT‐mediated DUTP nick end labelling (TUNEL) assay

Frozen sections of cardiac tissue were selected, and TUNEL staining was performed according to the manufacturer's guidelines using the TUNEL assay (C1090, Beyotime). The samples were examined under a fluorescence microscope. For each slide, five fields were randomly selected within a defined rectangular field area, and the number of positively stained cells was counted.

### 2.11 ASC oligomerization and fluorescence microscopy

To initiate the oligomerization of ASC, cells were subjected to treatment followed by lysis using NP‐40 lysis buffer (P0013F) obtained from Beyotime. The lysate was agitated on a shaker for 15 min at 4°C and then centrifuged at 6000 g for 15 min. The resulting cell pellets underwent cross‐linking with freshly prepared disuccinimidyl suberate (2 mM) for a duration of 30 min at 37°C before being collected through an additional centrifugation step. Subsequently, the pellets were resuspended in 5X SDS sample buffer and analysed via western blotting.

ASC speck analysis was conducted using fluorescence microscopy. Cells were fixed with 4% paraformaldehyde and permeabilized using 0.1% Triton X‐100. Nonspecific binding sites were blocked with 5% BSA, after which cells were incubated overnight at 4°C with ASC antibody (1:200, 67824S, Cell Signaling Technology). Detection was achieved using an Alexa Fluor 594‐labeled secondary antibody (1:200, 33112ES60, LABSELECT). Additionally, nuclei were stained with DAPI to facilitate counterstaining. Microscopic fields of view were captured randomly by a blinded experimenter employing laser confocal microscopy (spin SR model, OLYMPUS).

### 2.12 Co‐immunoprecipitation (Co‐IP) assays

Proteins were extracted from primary cardiomyocytes (1 × 10^7^) and animal myocardial tissue (10 mg) using 0.4 mL of RIPA buffer. The protein lysates were subsequently incubated with the primary antibody overnight at 4°C, while a portion of the lysate was reserved as an input sample. Following this, the protein lysates underwent precipitation utilizing protein G‐Sepharose beads at 4°C for a duration of 6–12 h. After washing with PBS, the protein G‐Sepharose beads were prepared for western blot analysis.

### 2.13 Co‐IP assays combined with liquid chromatography tandem mass spectrometry analysis

Cardiomyocytes were transfected with Flag‐USP20 plasmids prior to exposure to LPS/Nig treatment. Anti‐Flag antibodies and protein G‐Sepharose beads were subsequently added to the cell samples for co‐IP. The bound proteins were extracted from the co‐IP beads using SDT lysis buffer. Proteins were digested into peptides employing the filter‐aided sample preparation (FASP) method. Finally, liquid chromatography tandem mass spectrometry (LC‐MS/MS) analysis was conducted by PTM Bio Co., Ltd.

### 2.14 qPCR analysis

Total RNA was extracted from the cells using Trizol reagent (15596018CN, Thermo Fisher Scientific). cDNA synthesis was conducted with a Reverse Transcription Kit (Vazyme, R333‐01), and quantitative PCR analysis was performed utilizing SYBR Green reagent (Takara, DRR037A). β‐actin served as an internal control. The sequences of all primers are provided in Table S1.

### 2.15 Single‐cell RNA sequencing (scRNA‐seq)

For scRNA‐seq, cardiac tissues were harvested from both sham and CLP‐treated mice, followed by dissociation into individual cells using an appropriate dissociation solution. For each experimental group, single‐cell suspensions derived from 3 to 4 hearts were pooled to create a unified sample. The resulting single‐cell suspensions were then introduced onto the 10X Chromium platform for the capture of individual cells utilizing the 10X Genomics Chromium Single‐Cell 3′ kit. cDNA amplification, library construction and sequencing procedures were conducted by LC‐BIO Technologies Co., Ltd.

### 2.16 GEO database

The high‐throughput transcriptome sequencing data presented in the study are deposited in the GEO under accession code: GSE53007, GSE142615, and GSE153086.The data were standardized and differential gene expression analysis was performed using GEO2R. We selected Dubs with a correction adjusted *p*‐value < 0.05.

### 2.17 Statistical analysis

Statistical analyses were performed using GraphPad Prism 8.0 software (GraphPad). Descriptive statistics are presented as mean ± standard deviation (SD). The Shapiro–Wilk test was employed to assess the normality of each dataset. Comparisons between two groups were made using an independent samples *t*‐test, under the assumption that the data followed a normal distribution. For comparisons involving three or more groups, one‐way analysis of variance (ANOVA) was conducted, followed by Tukey's post‐hoc tests for further analysis. Survival data were analysed using the Kaplan–Meier method, and the log‐rank test was applied to compare the survival curves. *p* ˂ 0.05 was considered statistical significance.

## 3 RESULTS

### 3.1 Identification of USP20 as an essential contributor to septic cardiomyopathy

**FIGURE 1:**
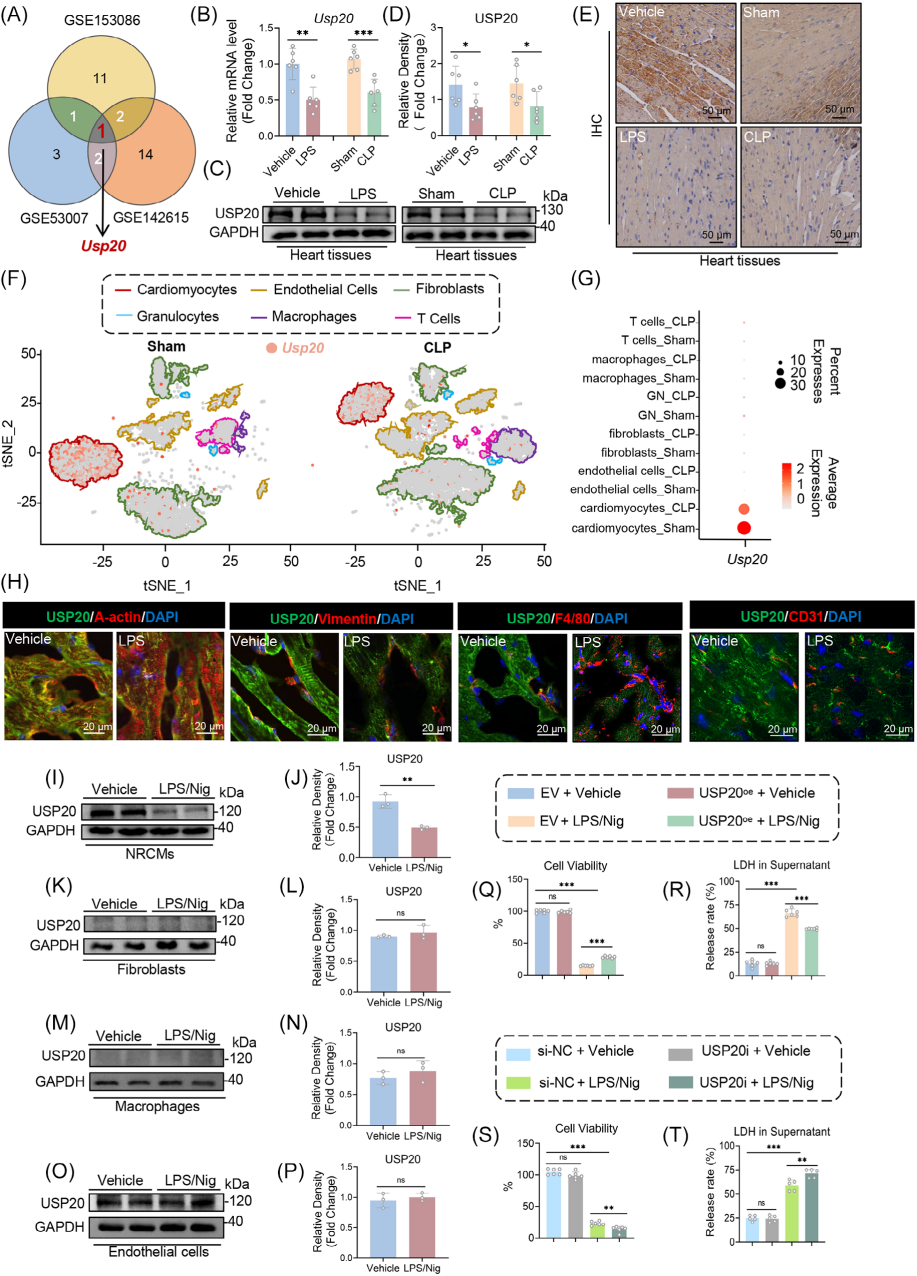
Identification of USP20 as an essential contributor to septic cardiomyopathy. (A) Transcriptomic analysis of deubiquitinating enzymes (DUBs) mRNA expression levels in the cardiac tissues of septic cardiomyopathy. The data were sourced from the GSE153086, GSE53007 and GSE142615 datasets. (B) The mRNA expression levels of *Usp20* in heart tissue of mice induced by lipopolysaccharide (LPS) and cecal ligation and puncture (CLP) respectively assessed by PCR. *n* = 6. (C‐D) The protein expression level of USP20 in heart tissue of mice induced by LPS and CLP respectively tested by western blotting (C) and the statistical results (D). *n* = 6. (E) Representative images of USP20 immunofluorescence staining of heart tissue from mice induced by LPS and CLP respectively. (F‐G) Single‐cell sequencing analysis of *Usp20* expression in heart tissue from control and CLP‐induced mice. For each group, single‐cell suspensions from 3 hearts were pooled as 1 sample. The tSNE dimensional reduction showing 6 main cell types of heart, including cardiomyocytes, endothelial cells, fibroblasts, granulocytes, macrophages and T cells. The red dots represent *Usp20* (F). Expression patterns of *Usp20* in individual cells from sham and CLP groups (G). (H) Representative immunofluorescence images exhibiting the colocalization of USP20 and A‐actin, USP20 and Vimentin, USP20 and F4/80 in heart tissues from both control and LPS‐induced mice. (I‐J) Expression of USP20 in neonatal rat cardiomyocytes (NRCMs) induced by LPS/nigericin (Nig) tested by western blotting (I) and the statistical results (J). *n *= 3. (K‐L) Expression of USP20 in primary fibroblasts induced by LPS/Nig tested by western blotting (K) and the statistical results (L). *n* = 3. (M‐N) Expression of USP20 in macrophages induced by LPS/Nig tested by western blotting (M) and the statistical results (N). *n *= 3. (O‐P) Expression of USP20 in endothelial cells induced by LPS/Nig tested by western blotting (O) and the statistical results (P). *n* = 3. (Q‐R) Cell viability (Q) and LDH release (R) of NRCMs overexpressing USP20 induced by LPS/Nig. *n* = 6. (S‐T) Cell viability (S) and LDH release (T) of USP20 knock down NRCMs induced by LPS/Nig. *n* = 6. Data are expressed as the mean ± standard deviation (SD). ***, *p* < 0.001; **, *p* < 0.01; *, *p *< 0.05, *p* > 0.05, ns: no differences.

We investigated the expression profiles of DUBs in septic cardiomyopathy utilizing data from the public GEO database (GSE153086, GSE53007 and GSE142615, Figure 1A). Notably, only USP20 mRNA exhibited a differential expression level among the three datasets. The mRNA and protein levels of USP20 in heart tissue were assessed, revealing a significant reduction in both mRNA and protein levels of USP20 in the hearts of mice induced by LPS and CLP (Figure 1B–D). IHC staining further confirmed that the expression of USP20 was decreased within the myocardium of these mice (Figure 1E). To explore the distribution of USP20 across various cellular components within myocardial tissue during sepsis, a single‐cell sequencing analysis was conducted. Using tSNE methodology, cells from control and CLP‐induced mouse heart tissues were classified into 6 distinct clusters. The localization of USP20 was predominantly observed in cardiomyocytes (Figure 1F,G). IF staining demonstrated that the reduced expression of USP20 primarily occurred in cardiomyocytes, while changes in its expression were less pronounced in fibroblasts, macrophages and endothelial cells in LPS‐induced septic cardiomyopathy (Figure 1H).

Our previous studies have demonstrated that pyroptosis plays a significant role in septic cardiomyopathy.^30^ Therefore, we employed LPS combined with Nig to stimulate cells, mimicking the progression of septic cardiomyopathy. Our findings indicated that the protein expression and mRNA level of USP20 significantly declined specifically in cardiomyocytes induced by LPS/Nig stimulation (Figure 1I,J). However, no significant alterations were detected within fibroblasts, macrophages or endothelial cells (Figures 1K–P and S1A–C). Therefore, the role of USP20 was investigated in cardiomyocytes following LPS/Nig injury in subsequent experiments. Overexpression and knockdown of USP20 in NRCMs were achieved through transfection with USP20 plasmid and siRNA (Figure S2A–D). The results indicated that overexpression of USP20 mitigated LPS/Nig‐induced injury in NRCMs (Figure 1Q,R), whereas depletion of USP20 aggravated the injury induced by LPS/Nig (Figure 1S,T). These findings suggest that USP20 is implicated in the pathogenesis of septic cardiomyopathy.

### 3.2 Cardiomyocyte‐specific USP20 deficiency aggravated myocardial injury and dysfunction in mice induced by LPS

**FIGURE 2:**
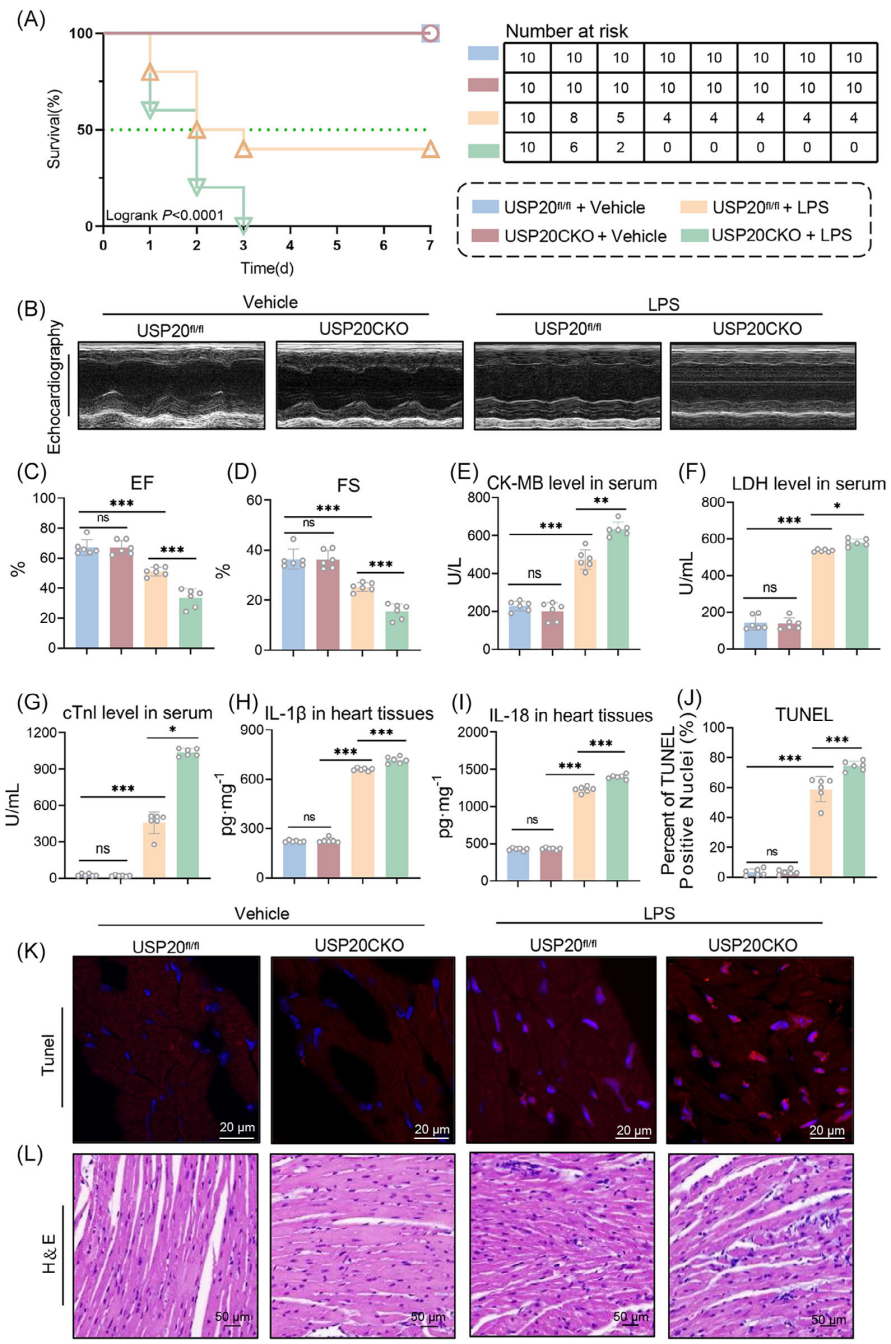
Cardiomyocyte‐specific USP20 deficiency aggravated myocardial injury and dysfunction in mice induced by LPS. (A) 7‐day survival rate of USP20^fl/fl^ and USP20^fl/fl^Myh6‐Cre (USP20CKO) mice induced by LPS. n = 10. (B‐C) Myocardial function parameters, ejection fraction (EF) (B) and fractional shortening (FS) (C) levels of USP20^fl/fl^ and USP20CKO mice induced by LPS measured by echocardiography. *n* = 6. (D) Representative echocardiography of mice from LPS model. (E‐G) The levels of creatine kinase isoenzyme MB (CK‐MB) (E), lactate dehydrogenase (LDH) (F) and cardiac troponin I (cTnI) (G) in serum of USP20^fl/fl^ and USP20CKO mice induced by LPS. *n* = 6. (H‐I) The levels of interleukin‐1β (IL‐1β) (H) and interleukin‐18 (IL‐18) (I) in serum of USP20^fl/fl^ and USP20CKO mice induced by LPS. *n* = 6. (J‐K) Representative images of heart tissue with TUNEL staining from USP20^fl/fl^ and USP20CKO mice induced by LPS (magnification × 400) (K) and statistical results (J). (L) Representative images of heart tissue with H&E staining from USP20^fl/fl^ and USP20CKO mice induced by LPS (magnification × 400). Data are expressed as the mean ± SD. ***, *p* < 0.001; **, *p* < 0.01; *, *p* < 0.05, *p* > 0.05, ns: no differences.

To elucidate the role of USP20 in cardiomyocytes during septic myocardial injury, the USP20CKO mice (Figure S3A–D) were constructed. Mouse models of septic cardiomyopathy were established through intraperitoneal injection of LPS. The effects of USP20 on LPS‐treated mice were evaluated by assessing the 7‐day survival rate, myocardial function, as well as markers of myocardial injury and inflammatory cytokines. Our findings revealed a significant decrease in the survival rate of LPS‐induced USP20CKO mice (Figure 2A). LPS‐treated mice exhibited reduced EF and FS levels, along with elevated serum levels of cTnI, CK‐MB, and LDH, as well as increased levels of the inflammatory cytokines IL‐1β and IL‐18. Notably, USP20 deficiency in heart tissue further exacerbated myocardial injury and dysfunction (Figure 2B–G and Table S2), alongside increased serum concentrations of inflammatory cytokines IL‐1β and IL‐18 (Figure 2H,I). Furthermore, TUNEL staining in heart tissue was performed in conjunction with H&E staining. Administration of LPS resulted in cardiomyocyte swelling and disarrangement while leading to an increase in cell death rates. Importantly, the absence of USP20 amplified these pathological alterations (Figure 2 J–L). No significant differences were observed in the aforementioned indicators between USP20^fl/fl^ mice and USP20CKO mice in the absence of LPS stimulation.

### 3.3 Cardiomyocyte‐specific USP20 knockout aggravated myocardial injury and dysfunction in CLP‐induced mice

**FIGURE 3:**
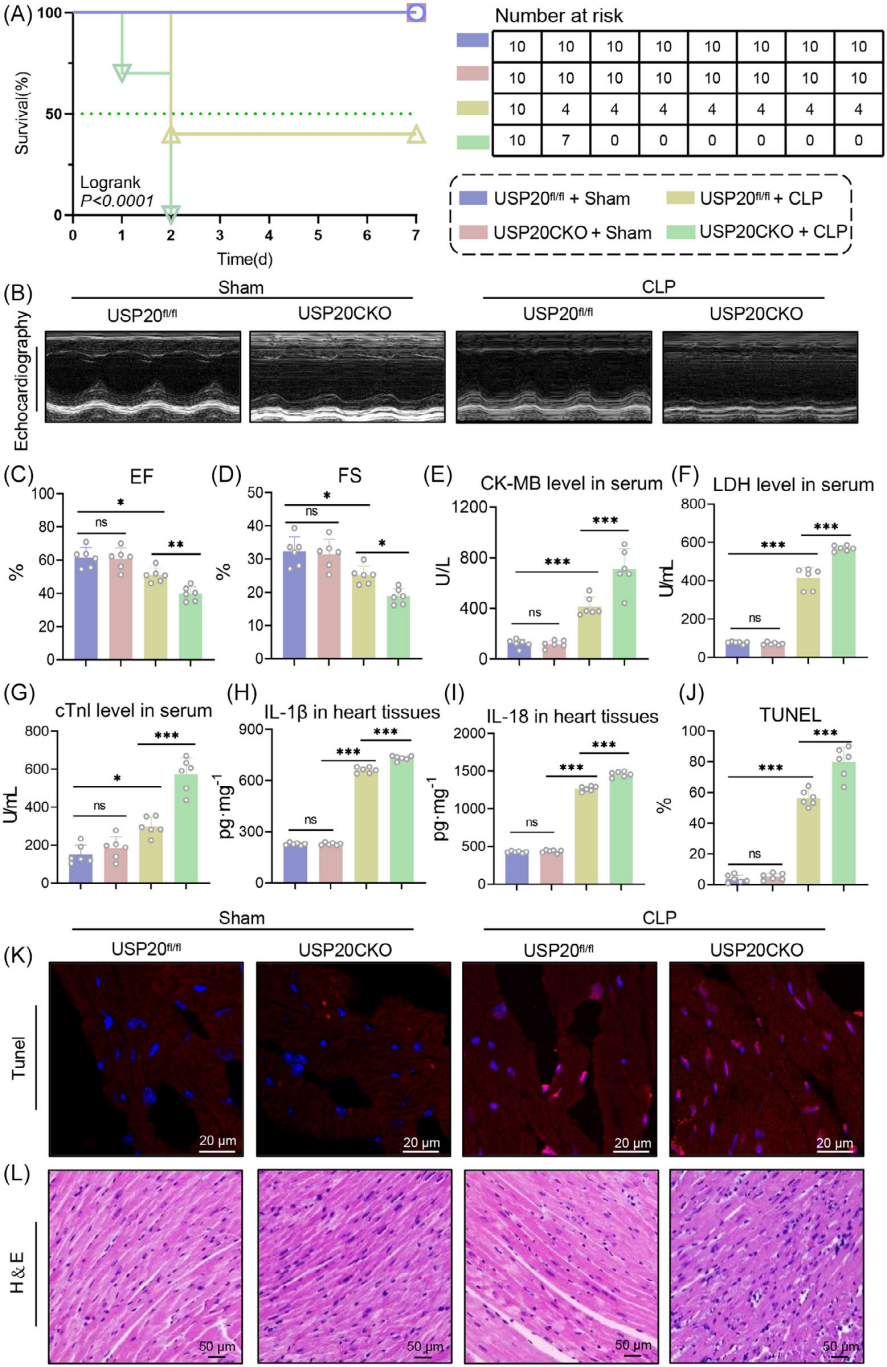
Cardiomyocyte‐specific USP20 knockout aggravated myocardial injury and dysfunction in CLP‐induced mice. (A) 7‐day survival rate of USP20^fl/fl^ and USP20CKO mice induced by CLP. *n* = 10. (B‐C) The levels of EF (B) and FS (C) of USP20^fl/fl^ and USP20CKO mice induced by CLP measured by echocardiography. n = 6. (D) Representative echocardiography of mice from CLP model. (E‐G) The levels of CK‐MB (E), LDH (F) and cTnI (G) in serum of USP20^fl/fl^ and USP20CKO mice induced by CLP. *n* = 6. (H‐I) The levels of IL‐1β (H) and IL‐18 (I) in serum of USP20^fl/fl^ and USP20CKO mice induced by CLP. *n* = 6. (J‐K) Representative images of heart tissue with TUNEL staining from USP20^fl/fl^ and USP20CKO mice induced by CLP (magnification × 400) (K) and statistical results (J). (L) Representative images of heart tissue with H&E staining from USP20^fl/fl^ and USP20CKO mice induced by CLP (magnification × 400). Data are expressed as the mean ± SD. ***, *p* < 0.001; *, *p* < 0.05, *p* > 0.05, ns: no differences.

To confirm the role of USP20 in septic cardiomyopathy, a mouse model of CLP was established. The results mirrored those from the LPS model. CLP administration significantly reduced the 7‐day survival rates and left ventricular systolic and diastolic function in mice. While these were worsened in CLP‐induced USP20CKO mice (Figure 3A–D and Table S3). The levels of myocardial injury marker including cTnI, CK‐MB and LDH, as well as inflammatory cytokines IL‐1β and IL‐18 in serum exhibited a similar trend (Figure 3E–I). Analysis of TUNEL and H&E staining indicated that cardiomyocyte‐specific USP20 deficiency intensified CLP‐induced cardiomyocyte disarray and death (Figure 3J–L). These findings highlight that USP20 deficiency exacerbates myocardial injury and dysfunction in septic mice.

### 3.4 Identification of NLPR3 as the substrate protein of USP20

**FIGURE 4:**
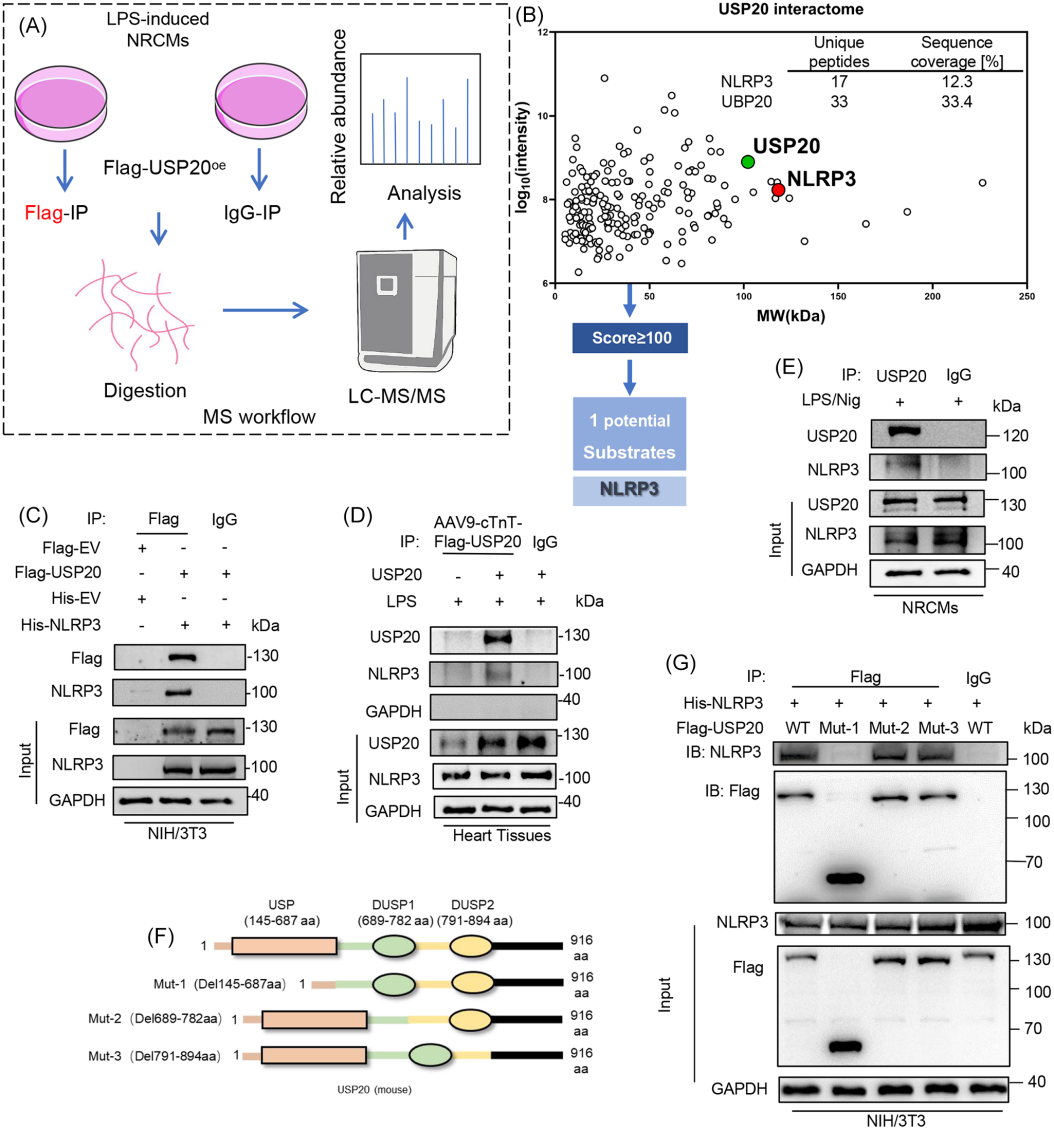
Identification of NLPR3 as the substrate protein of USP20. (A) The workflow for USP20 substrate screening. The candidate substrates of USP20 screened by the liquid chromatography tandem mass spectrometry (LC‐MS/MS). (B) A two‐dimensional (2D) plot is presented, illustrating the log_10_ signal intensity of quantified proteins on the *y*‐axis (indicative of enrichment in Flag‐USP20 immunoprecipitation) and the molecular weight (MW) of these proteins on the *x*‐axis. Additionally, a table lists the candidate substrates for USP20. (C) Co‐immunoprecipitation (Co‐IP) of USP20 and NLRP3 in NIH/3T3 cells co‐transfected with plasmids encoding Flag‐USP20 and His‐NLRP3. Exogenous USP20 was immunoprecipitated by anti‐Flag antibody. (D) Co‐IP of USP20 and NLRP3 in heart tissue of mice induced by LPS. Endogenous USP20 was immunoprecipitated by anti‐USP20. (E) Co‐IP of USP20 and NLRP3 in NRCMs induced by LPS/Nig. Endogenous USP20 was immunoprecipitated by anti‐USP20. (F) Schematic illustration of the USP20 domain. (G) Co‐IP of Flag‐WT‐USP20, Flag‐mut‐USP20 and His‐NLRP3 in NIH/3T3 cells co‐transfected with plasmids of Flag‐WT‐USP20, Flag‐mut‐USP20 and His‐NLRP3. Exogenous WT or mutated USP20 was immunoprecipitated by anti‐Flag antibody. WT, wild type.

USP20 functions as a deubiquitinating enzyme that modulates protein activity by regulating the ubiquitination of substrate proteins. To identify the substrate molecules targeted by USP20 in the septic cardiomyopathy, co‐IP conjunction with LC‐MS/MS were employed in subsequent experiments (Figure 4A). After eliminating the peptides linked to both the light and heavy chains of the antibody, we identified a single potential USP20 target protein with a score exceeding 100, namely NLRP3. It was determined that NLRP3 is closely associated with the pathogenesis of septic cardiomyopathy. Consequently, NLRP3 was recognized as a potential substrate protein of USP20 (Figure 4B). The interaction between USP20 and NLRP3 was subsequently investigated. Co‐transfection of USP20 and NLRP3 plasmids into NIH/3T3 cells yielded results from co‐IP experiments, which demonstrated a direct interaction between USP20 and NLRP3 (Figure 4C). These findings were corroborated in myocardial tissue and cardiomyocytes, confirming the binding of USP20 to NLRP3 (Figure 4D,E). The USP20 protein comprises three distinct structural domains: the ubiquitin‐specific protease domain (USP), dual‐specificity phosphatase 1 (DUSP1), and dual‐specificity phosphatase 2 (DUSP2) (Figure 4F). Subsequently, mutated plasmids of USP20 targeting different regions were constructed and transfected into NIH/3T3 cells. Co‐IP experiments conducted with these mutants revealed that USP20 interacts with NLRP3 through its USP domain (Figure 4G).

### 3.5 USP20 affected septic cardiomyopathy by regulating NLRP3 activity

**FIGURE 5:**
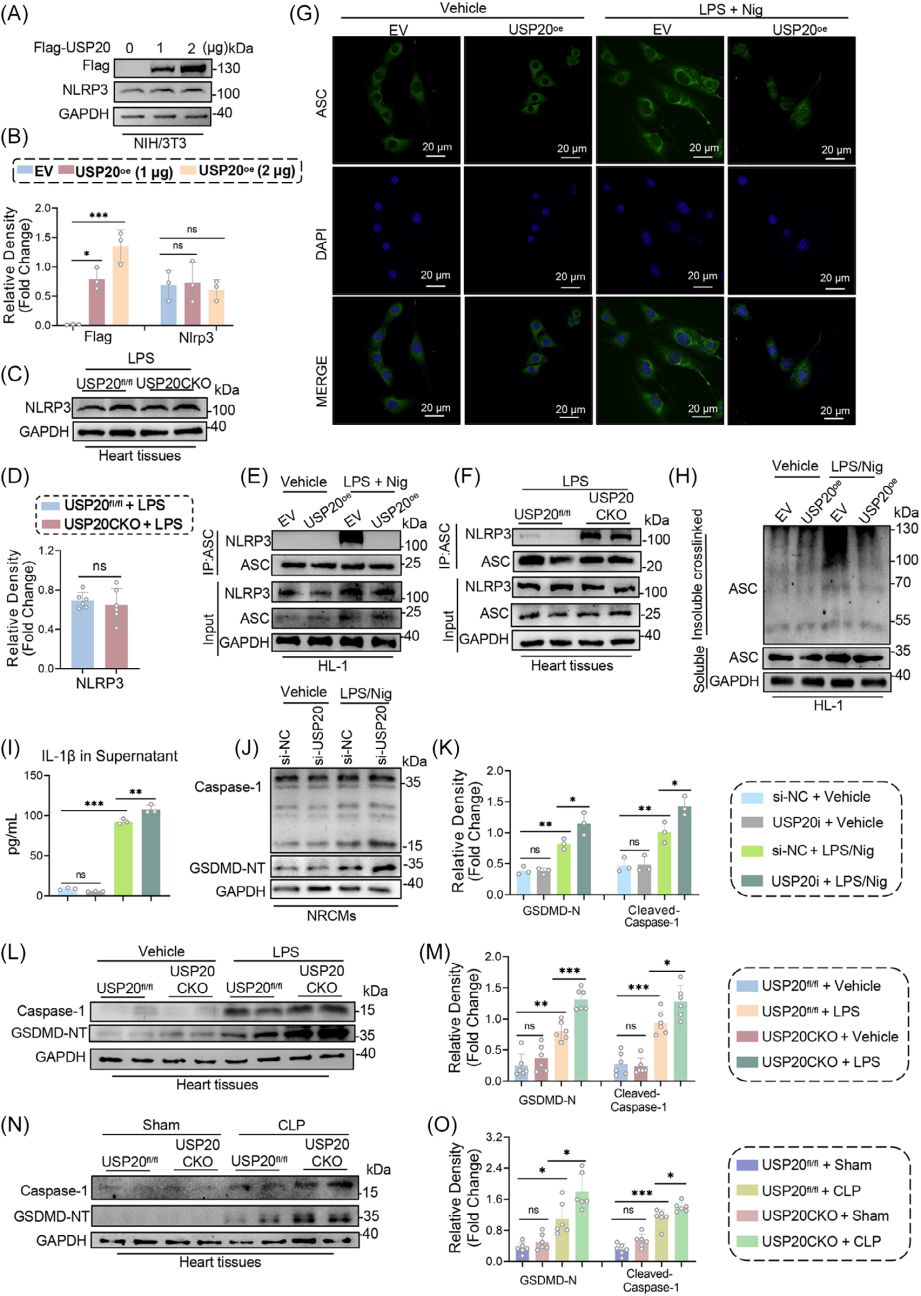
USP20 affected septic cardiomyopathy by regulating NLRP3 activity. (A‐B) Expression of USP20 and NLRP3 protein in NIH/3T3 cells transfected with different doses of Flag‐USP20 plasmids (A) and statistical results (B). *n* = 3. (C‐D) Expression of NLRP3 protein in heart tissue of USP20^fl/fl^ and USP20CKO mice induced by LPS (C) and statistical results (D). *n* = 6. (E) Co‐IP of endogenous ASC and NLRP3 in HL‐1 cells overexpressing USP20 induced by LPS/Nig. Endogenous ASC was immunoprecipitated by anti‐ASC antibody. (F) Co‐IP of endogenous ASC and NLRP3 in heart tissues from USP20^fl/fl^ and USP20CKO mice induced by LPS. Endogenous ASC was immunoprecipitated by anti‐ASC antibody. (G) Immunofluorescence staining of ASC in cells overexpressing USP20 induced by LPS/Nig (scale bar, 20 µm). (H) Co‐IP of ASC was performed on both chemically cross‐linked NP‐40 insoluble and NP‐40 soluble fractions obtained from HL‐1 cell lysates. NRCMs were subjected to treatment with LPS/Nig in order to establish a cardiomyocyte injury model, following transfection with either USP20 small interfering RNA (si‐USP20) or negative control small interfering RNA (si‐NC). (I) IL‐1β level in supernatants of NRCMs from each group. *n *= 3. (J‐K) Protein expressions level of Caspase‐1 and GSDMD‐NT in NRCMs (J) and statistical results (K). *n* = 3. (L‐M) Expressions of Caspase‐1 and GSDMD‐NT proteins in heart tissue of USP20^fl/fl^ and USP20CKO mice induced by LPS (L) and statistical results (M). *n* = 6. (N‐O) Expressions of Caspase‐1 and GSDMD‐NT proteins in heart tissue of USP20^fl/fl^ and USP20CKO mice induced by CLP (N) and statistical results (O). *n *= 6. Data are expressed as the mean ± SD. ***, *p* < 0.001; **, *p* < 0.01; *, *p* < 0.05, *p* > 0.05, ns: no differences.

The subsequent investigation concentrated on the regulatory role of USP20 in the NLRP3 protein. DUBs typically preserve the stability of substrate proteins by removing ubiquitin from these proteins. Various concentrations of Flag‐USP20 plasmids were transfected into NIH/3T3 cells to assess NLRP3 expression levels. Notably, we observed that, despite an increase in USP20 expression, there was no significant change in NLRP3 levels (Figure 5A,B). In addition, we did not observe any alterations in the degradation rate of NLRP3 in 3T3 cells treated with cycloheximide (CHX) following either the interference or overexpression of USP20 (Figure S4A–D). These findings were further corroborated utilizing USP20CKO mice, which demonstrated that NLRP3 expression in myocardial tissue remained unchanged under LPS induction conditions (Figure 5C,D). We hypothesized that USP20 might be involved in modulating the activation of NLRP3. The recruitment of NLRP3 to ASC, forming the NLRP3 inflammasome complex, represents a critical step for its activation.^31^ Our results indicated that overexpression of USP20 notably diminished the interaction between NLRP3 and ASC within HL‐1 cells stimulated by LPS/Nig (Figure 5E). In contrast, knockout of USP20 resulted in increased binding between NLRP3 and ASC within heart tissues of LPS‐induced mice (Figure 5F). Moreover, immunofluorescence assays revealed a substantial rise in ASC speck formation following treatment with LPS/Nig. However, this phenomenon was significantly mitigated through overexpression of USP20 (Figure 5G). To further verify these observations, lysates from HL‐1 cells were subjected to bifunctional chemical cross‐linking followed by ASC immunoblotting analysis. Consistent with previous findings, oligomerization of ASC induced by LPS/Nig was attenuated upon overexpression of USP20 (Figure 5H). Additionally, we found that challenging NRCMs with LPS/Nig resulted in an elevated level of IL‐1β in the supernatant. Notably, the deficiency of USP20 further augmented the IL‐1β levels in the supernatant (Figure 5I). Similarly, USP20 deficiency exacerbated the increased protein levels of Caspase‐1 and N‐terminal fragment of GSDMD (GSDMD‐NT) induced by LPS/Nig (Figures 5J,K). In contrast, overexpression of USP20 effectively reduced the IL‐1β level in the supernatant (Figure S5A) and reversed the expression of Caspase‐1 and GSDMD‐NT induced by LPS/Nig (Figure S5B,C). Moreover, we substantiated these findings using USP20CKO mice. The results indicated that mice induced with LPS displayed elevated levels of Caspase‐1 and GSDMD‐NT proteins in heart tissue. However, cardiomyocyte‐specific USP20 deficiency led to an even greater increase in the expression of these proteins within cardiac tissue (Figure 5L,M). Consistent results were observed following CLP administration (Figure 5N,O). These findings suggest that USP20 ameliorates septic myocardial injury and dysfunction by regulating NLRP3‐mediated pyroptosis.

### 3.6 USP20 deubiquitinated NLRP3 by removing K63‐linked ubiquitin at the K243 residue via its active site C154

**FIGURE 6:**
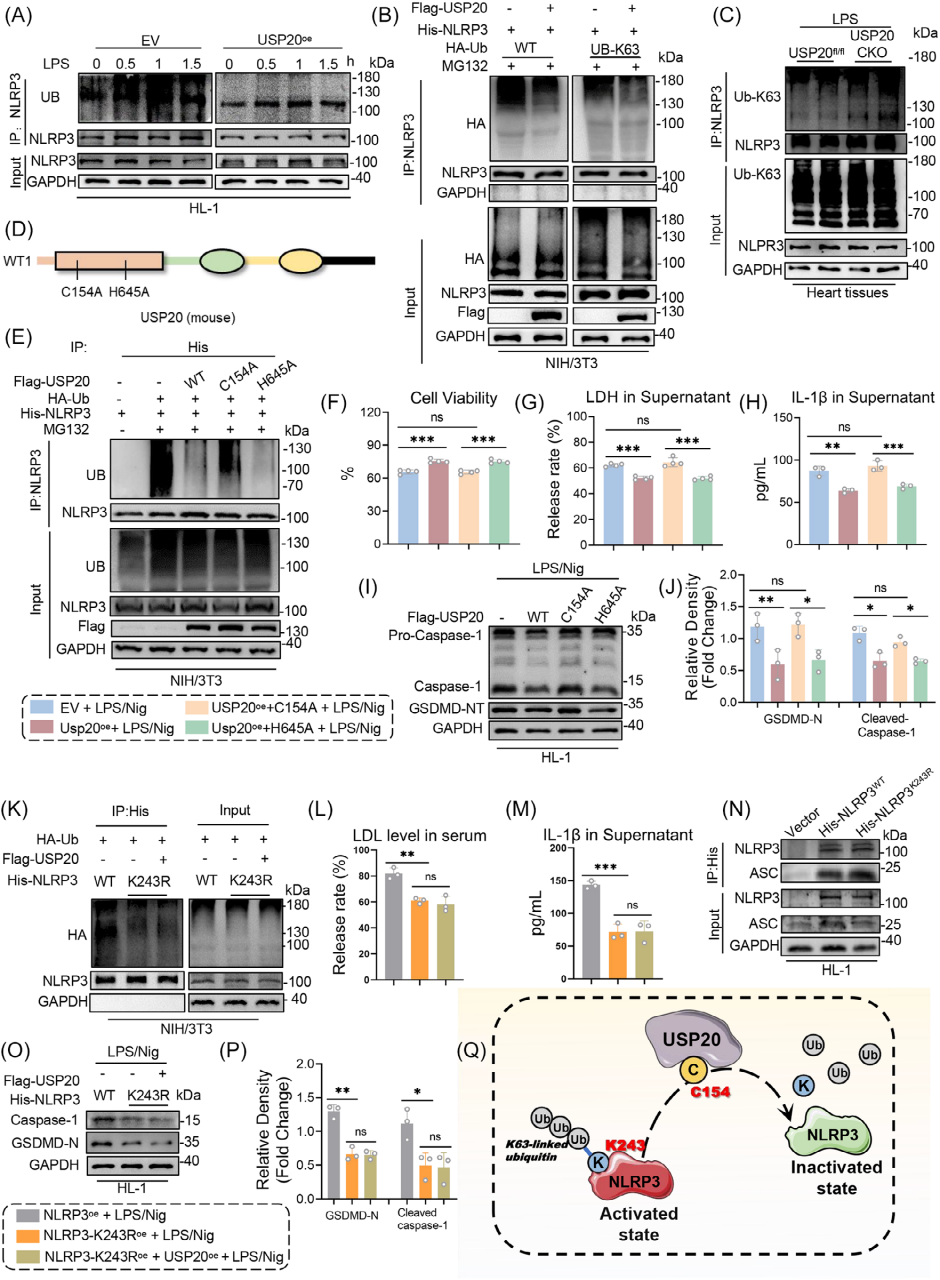
USP20 deubiquitinated NLRP3 by removing K63‐linked ubiquitin at the K243 residue via its active site C154. (A) NLRP3 was immunoprecipitated from HL‐1 cells co‐transfected with either an empty vector (EV) or a USP20 overexpression plasmid (USP20^oe^), followed by exposure to lipopolysaccharide (LPS, 1 µg/mL) in a pulse‐chase assay. The ubiquitination status of NLRP3 was assessed through immunoblotting utilizing a ubiquitin‐specific antibody, aimed at elucidating the ubiquitination patterns influenced by USP20. (B) Immunoprecipitation of NLRP3 was performed in NIH/3T3 cells co‐transfected with His‐NLRP3, HA‐ubiquitin (UB), and HA‐K63‐UB plasmids before MG132 treatment (10 µM). The ubiquitinated NLRP3 was detected by immunoblotting using a His‐specific antibody to elucidate the ubiquitination patterns regulated by USP20. (C) Immunoprecipitation of NLRP3 in heart tissues of USP20^fl/fl^ and USP20CKO mice induced by LPS. Ubiquitinated NLRP3 was assessed through immunoblotting using an UB‐K63 antibody to elucidate the K63 ubiquitination level of NLRP3 regulated by USP20. (D) Schematic illustration of the active site of USP20. (E) Immunoprecipitation of NLRP3 in NIH/3T3 cells that co‐transfected with plasmids encoding His‐NLRP3, HA‐UB, USP20‐WT, USP20‐C154A and USP20‐H645A plasmids prior to treatment with MG132 (10 µM). The ubiquitinated form of NLRP3 was detected through immunoblotting utilizing a His‐specific antibody to clarify the ubiquitination level of NLRP3 regulated by the active site of USP20. (F‐H) Cell viability (F), LDH release (G), and IL‐1β in supernatant (H) of LPS/Nig‐induced cells overexpressing USP20‐WT, USP20‐C154A and USP20‐H645A plasmids. *n* = 3. (I‐J) Expressions of Caspase‐1 and GSDMD‐NT protein in LPS/Nig‐induced HL‐1 cells overexpressing USP20‐WT, USP20‐C154A and USP20‐H645A plasmids (I) and statistical results (J). *n* = 3. (K) Immunoprecipitation of NLRP3 in NIH/3T3 cells that co‐transfected with plasmids encoding HA‐UB, His‐NLRP3‐WT, and His‐NLRP3‐K243R. Ubiquitinated NLRP3 was detected by immunoblotting via using an His‐specific antibody to clarify the ubiquitination lysine residues of NLRP3 regulated USP20. (L‐M) LDH release (L) and IL‐1β level in supernatant (M) of LPS/Nig‐induced cells overexpressing NLRP3‐WT, NLRP3‐K243R and USP20. *n* = 3. (N) Co‐IP of endogenous ASC and NLRP3 in HL‐1 cells overexpressing NLRP3‐WT, NLRP3‐K243R and USP20. Endogenous ASC was immunoprecipitated by anti‐ASC antibody. (O‐P) Expressions of Caspase‐1 and GSDMD‐NT protein in HL‐1 cells overexpressing NLRP3‐WT, NLRP3‐K243R and USP20 (O) and statistical results (P). *n* = 3. (Q) Schematic illustration of NLRP3 deubiquitinated by USP20. Data are expressed as the mean ± SD. ***, *p* < 0.001; **, *p* < 0.01; *, *p* < 0.05, *p* > 0.05, ns: no differences.

Having elucidated the role of USP20 in modulating NLRP3 activity, we proceeded to investigate the specific types of ubiquitin (UB) linkages associated with NLRP3 that are deubiquitinated by USP20, as well as the particular sites of ubiquitination involved. The results demonstrated that overexpression of USP20 led to a significant reduction in the amount of UB attached to NLRP3 (Figure 6A). Among various forms, K63‐linked ubiquitin chains are one of the most extensively studied.^32^, ^33^, ^34^, ^35^ Consequently, our focus for this study was on detecting K63‐linked ubiquitin chains. The findings indicated that overexpression of USP20 resulted in a marked decrease in ubiquitin molecules associated with NLRP3 in cells transfected with either wild‐type UB plasmid or a plasmid retaining only K63 activity (UB‐K63) (Figure 6B). This result was further validated using LPS‐induced USP20CKO mice, which exhibited an increase in K63‐linked ubiquitin attachment to NLRP3 due to USP20 deficiency (Figure 6C). These findings suggest that USP20 specifically removes K63‐linked ubiquitination from the NLRP3 protein. We next examined the active site through which USP20 exerts its function. The cysteine residue at position 154 (C154) and histidine residue at position 645 (H645) were supposed as catalytic residues essential for the deubiquitinating function of USP20 (Figure 6D). Accordingly, mutant USP20 plasmids with C154A (mutation of cysteine to alanine at C154) or H645A (mutation of histidine to alanine at H645) were constructed. Both USP20^C154A^ and USP20^H645A^ mutants retained the ability to bind to NLRP3 (Figure S6). However, the ability of USP20^C154A^ to remove ubiquitin molecules from NLRP3 was impaired, whereas no decrease in the activity of deubiquitination was observed for USP20^H645A^ (Figure 6E). Furthermore, in comparison to the group overexpressing USP20^WT^, the overexpression of USP20^C154A^ did not demonstrate a significant reduction in cell injury, supernatant LDH release, or IL‐1β concentration induced by LPS/Nig (Figure 6F–H). Additionally, USP20^C154A^ appeared to lose its regulatory effect on NLRP3 activation, as indicated by the upregulation of Caspase‐1 and GSDMD‐NT expression following LPS/Nig treatment (Figure 6I,J). These findings suggest that the C154 site of USP20 plays a crucial role in the deubiquitination process of NLRP3.

In addition, the site of NLRP3 deubiquitination by USP20 was identified. The residue K243 in the NLRP3 protein was selected as a candidate for this modification. Mutants of NLRP3 were generated by substituting lysine with arginine at position K243 (NLRP3^K243R^). A co‐IP‐based ubiquitinated peptide enrichment analysis was performed. The results indicated a significant reduction in the polyubiquitination of NLRP3^K243R^, which was not further decreased upon overexpression of USP20 (Figure 6K). When compared to NLRP3^WT^, NLRP3^K243R^ exhibited reduced LDH release and lower supernatant levels of IL‐1β in HL‐1 cells following LPS/Nig injury. Notably, overexpression of USP20 did not lead to an additional decrease in LDH release or IL‐1β levels (Figure 6L,M). The co‐IP experiments involving NLRP3 and ASC demonstrated that the binding of ASC to NLRP3^K243R^ under LPS/Nig stimulation was reduced (Figure 6N). Furthermore, expression levels of Caspase‐1 and GSDMD‐NT induced by LPS/Nig were diminished in the presence of NLRP3^K243R^, while overexpression of USP20 did not confer any additional protective effects (Figure 6O,P). These findings suggest that K243 is essential for USP20‐mediated deubiquitination of NLRP3 and subsequent activation of the NLRP3 inflammasome. Thus, it can be concluded that USP20 interacts with K243 of NLRP3 through its active site C154 to inhibit the activation of NLRP3 within cardiomyocytes (Figure 6Q).

### 3.7 Overexpression of USP20 improved septic cardiomyopathy induced by LPS

**FIGURE 7:**
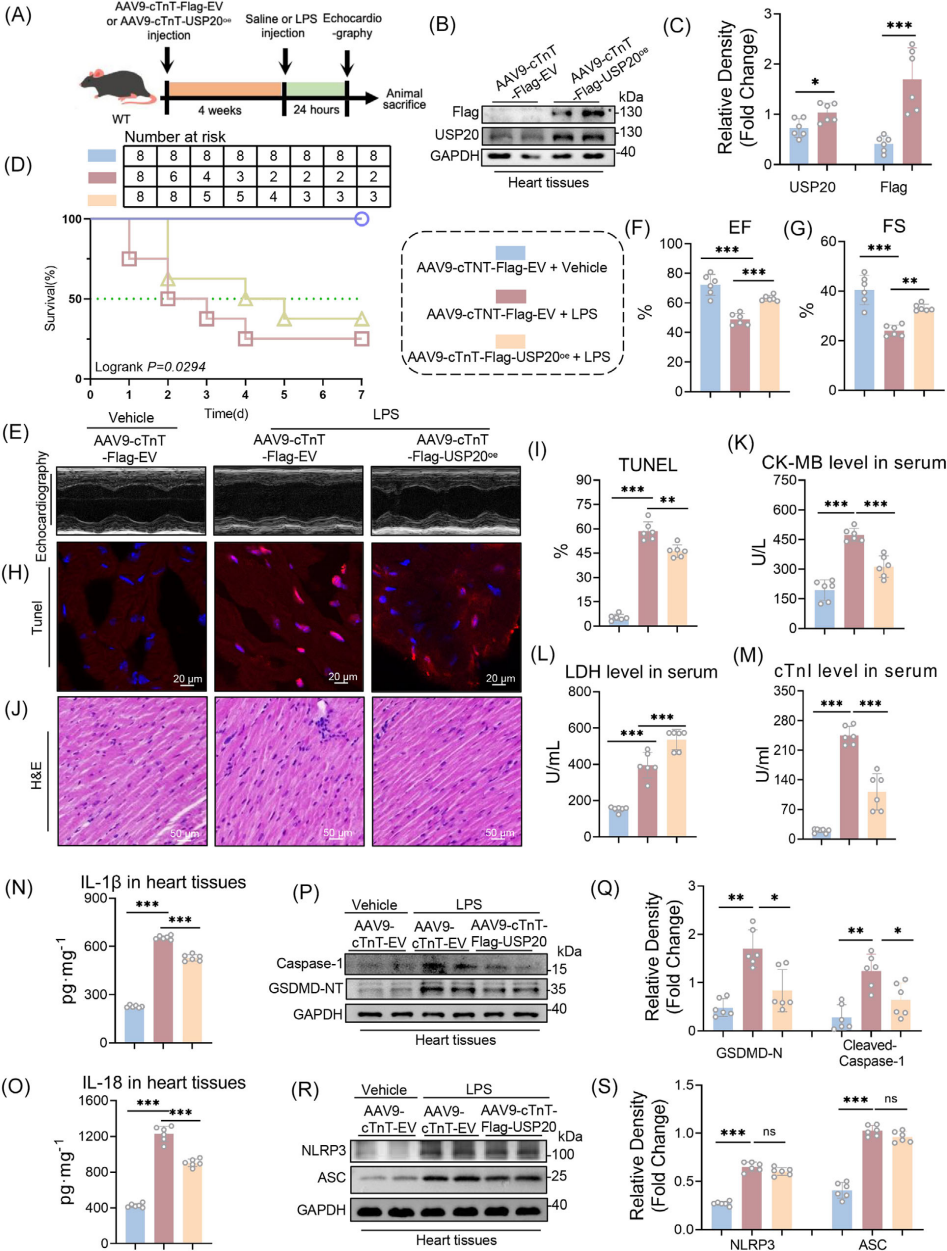
Overexpression of USP20 improved septic cardiomyopathy induced by LPS. (A) Schematic diagram of LPS‐induce septic myocardial injury mouse model in mice overexpressing USP20 in cardiomyocytes. (B‐C) Expressions of Flag‐USP20 protein in heart tissue of mice (B) and statistical results (C). n = 3. (D) 7‐day survival rate of mice from each group. *n *= 10. (E) Representative echocardiography of mice from LPS model. (F‐G) The EF (F) and FS (G) levels of mice induced by LPS. *n* = 6. (H‐I) Representative images of heart tissue with TUNEL staining from mice of each group (magnification × 400) (H) and statistical results (I). (J) Representative images of heart tissue with H&E staining from mice of each group (magnification × 400). (K‐M) The levels of CK‐MB (K), LDH (L) and cTnI (M) in serum from mice of each group. *n* = 6. (N‐O) The levels of IL‐1β (N) and IL‐18 (O) in serum from mice of each group. n = 6. (P‐Q) Expressions of Caspase‐1 and GSDMD‐NT proteins in heart tissue of mice from each group (P) and statistical results (Q). *n* = 6. (R‐S) Expressions of NLRP3 and ASC proteins in heart tissue of mice from each group (R) and statistical results (S). *n* = 6. Data are expressed as the mean ± SD. ***, *p* < 0.001; **, *p* < 0.01; *, *p* < 0.05.

To further investigate the role of USP20 in septic cardiomyopathy, we introduced an AAV9 virus carrying USP20 cDNA to specifically overexpress USP20 in myocardial tissue (Figure 7A–C). Before validation in vivo, we examined the biosafety of potential side effects of AAV9‐cTnT‐USP20^oe^ delivery in mice. As shown in Figure S7A–I, the treatment with AAV9‐cTnT‐USP20^oe^ did not result in any significant impairment of liver function, kidney function, or lipid metabolism. Then, the protective effect of USP20 on mice subjected to LPS treatment was evaluated. Our findings indicated that LPS administration substantially decreased the 7‐day survival rate, EF and FS, which were improved in mice overexpressing USP20 in cardiomyocytes (Figure 7D–G and Table S4). Additionally, TUNEL and H&E staining analyses of heart tissue revealed that LPS exposure led to cell swelling, disorganized cellular architecture, and increased pyroptosis. These adverse effects were mitigated by cardiomyocyte‐specific USP20 overexpression (Figure 7H–J). Furthermore, we observed that the elevation in serum levels of cTnI, CK‐MB, and LDH induced by LPS was significantly reduced following USP20 overexpression (Figure 7K–M). Moreover, overexpression of USP20 effectively diminished the expression levels of Caspase‐1 and GSDMD‐NT proteins, as well as the serum levels of IL‐1β and IL‐18 induced by LPS treatment (Figure 7N–Q). However, the expression levels of NLRP3 and ASC proteins in the LPS‐induced murine myocardium remained unchanged following overexpression of USP20 (Figure 7R,S). These results suggest that upregulation of USP20 enhances outcomes in septic cardiac dysfunction and alleviates myocardial injury induced by LPS.

### 3.8 USP20 enhanced septic cardiomyopathy via NLRP3 dependency

**FIGURE 8:**
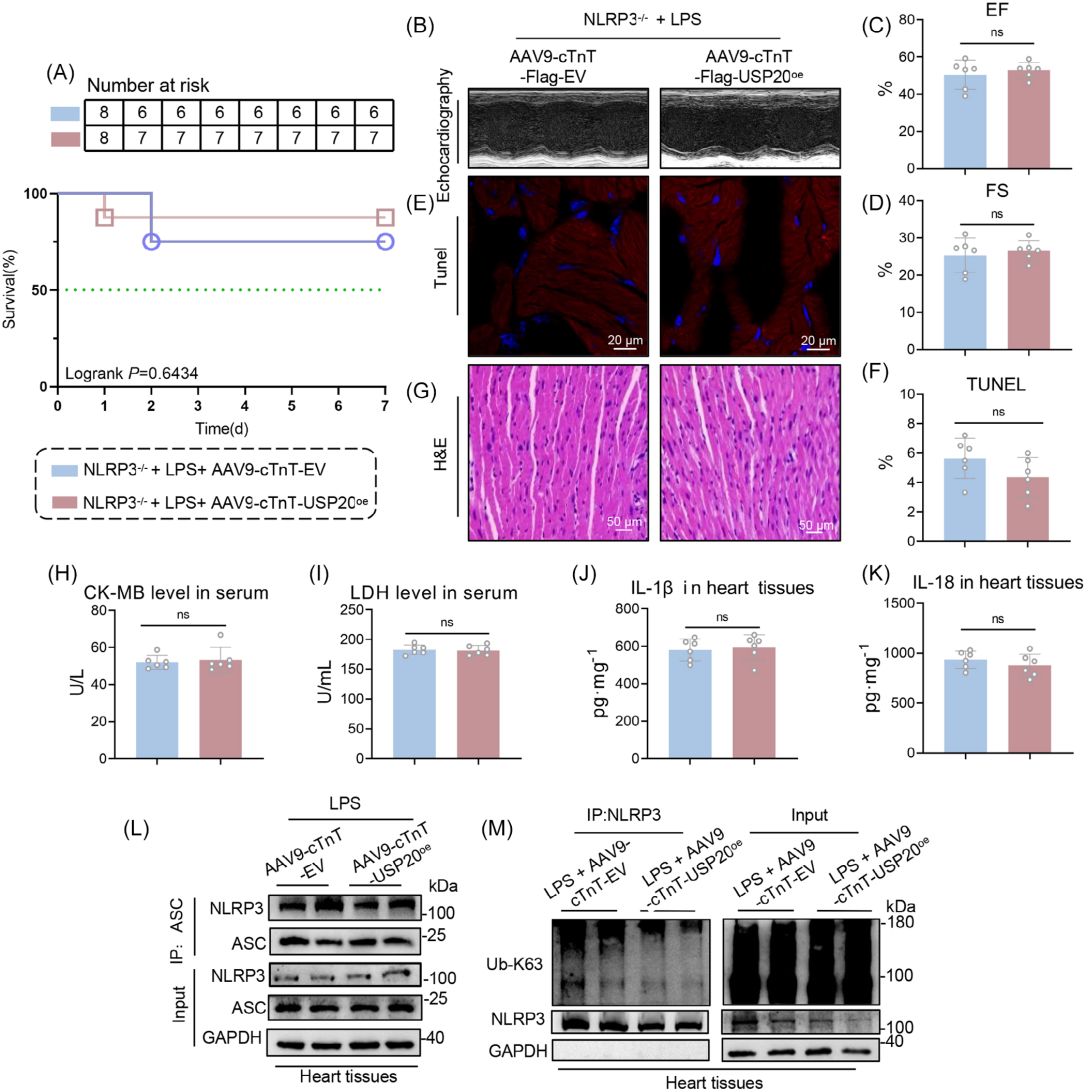
USP20 enhanced septic cardiomyopathy via NLRP3 dependency. (A) 7‐day survival rate of mice induced by LPS. n = 10. (B) Representative echocardiography of mice from LPS model. (C‐D) The EF (C) and FS (D) levels of mice induced by LPS. *n* = 6. (E‐F) Representative images of heart tissue with TUNEL staining from mice induced by LPS (magnification × 400) (E) and statistical results (F). (G) Representative images of heart tissue with H&E staining from mice induced by LPS (magnification × 400). (H‐I) The levels of CK‐MB (H) and LDH (I) in serum from mice induced by LPS. *n* = 6. (J‐K) The levels of IL‐1β (J) and IL‐18 (K) in serum from mice induced by LPS (*n* = 6). (L) Co‐IP of endogenous ASC and NLRP3 in heart tissues of LPS‐induced mice. Endogenous ASC was immunoprecipitated by anti‐ASC antibody. (M) Immunoprecipitation of NLRP3 in heart tissues of LPS‐induced mice. Ubiquitinated NLRP3 was assessed by immunoblotting using an UB‐K63 antibody. Data are expressed as the mean ± SD. *p* > 0.05, ns: no differences.

To validate the hypothesis that USP20 ameliorates septic cardiomyopathy through inhibiting NLRP3, we generated NLRP3^−/−^ mice with specific overexpression of USP20 in cardiac tissue by employing an AAV9 virus containing USP20 cDNA (Figure S8). The 7‐day survival rate and myocardial function assessments indicated by EF and FS revealed no significant differences between LPS‐induced NLRP3^−/−^ mice and those with overexpressing USP20 after LPS treatment (Figure 8A–D and Table S5). Additionally, the cellular morphology and proportion of TUNEL‐positive cells within myocardial samples demonstrated comparable results across both groups (Figure 8E–G). Furthermore, the serum levels of cardiac injury biomarkers, including CK‐MB and LDH, along with inflammatory cytokines IL‐1β and IL‐18 exhibited similar trends in their alterations (Figure 8H–K), suggesting that the cardioprotective effects afforded by USP20 against LPS‐induced myocardial dysfunction are negated upon knockout of NLRP3. At a mechanistic level, our observations indicated that overexpression of USP20 led to a reduction in K63‐linked polyubiquitination of NLRP3 within the heart tissue of LPS‐treated mice. This modification consequently inhibited the interaction between NLRP3 and ASC (Figure 8L,M).

## 4 DISCUSSION

Our findings indicated that USP20 was downregulated in the myocardial tissues of septic mice. Cardiomyocyte‐specific deficiency of USP20 exacerbated myocardial injury and cardiac dysfunction induced by LPS and CLP. Conversely, overexpression of USP20 in cardiomyocytes alleviated LPS‐induced myocardial damage. USP20 was identified as a significant regulator of pyroptosis triggered by LPS. Mechanistically, USP20 deubiquitinated NLRP3 by removing K63‐linked ubiquitin at the K243 residue through its active site C154. This action inhibited the interaction between NLRP3 and ASC, consequently suppressing NLRP3 activation and subsequent pyroptosis. These findings clearly demonstrate that USP20 plays a critical role in the pathogenesis of sepsis‐induced myocardial injury. A schematic diagram illustrating these principal findings is presented in the Graphic Abstract.

Ubiquitination serves as a crucial form of post‐translational modification of proteins. DUBs play essential roles by cleaving ubiquitin from substrate proteins, positioning them as key participants in the ubiquitination process.^36^ Growing evidence indicates that DUBs are implicated in the pathogenesis of cardiovascular diseases. For instance, it has been reported that USP28 deubiquitinates and stabilizes PPARα, thereby preventing mitochondrial morphological and functional defects while improving diabetic myocardial dysfunction.^20^ USP25 has been shown to alleviate cardiac hypertrophy through the stabilization of SERCA2a.^37^ Our findings revealed a reduction in USP20 levels in the hearts of mice induced by LPS and CLP. USP20 was identified as a key regulatory factor in LPS‐induced pyroptosis. The downregulation of USP20 in septic cardiomyopathy may be attributed to the following potential factors. Under sepsis conditions, the levels of pro‐inflammatory cytokines are markedly elevated. These cytokines may suppress the transcription of USP20 by activating downstream signalling pathways, thereby reducing the synthesis of the USP20 protein. Meanwhile, the inflammatory storm interferes the normal intracellular signal transduction and metabolic balance, indirectly affecting the expression and stability of USP20. Secondly, USP20 protein may be damaged by oxidative stress or ubiquitinated by other E3 ubiquitin ligases, which could accelerate its degradation. In the present study, NLPR3 has been identified as a substrate protein of USP20. The down‐regulation of USP20 results in impaired deubiquitination of NLRP3, thereby enhancing NLRP3 inflammasome activation and amplifying the inflammatory response. This process establishes a vicious cycle that further exacerbates myocardial injury and dysfunction.

The degradation and activation of NLRP3 can be modulated through deubiquitination modifications.^38^ NLRP3 deubiquitination is recognized as a critical step in the activation of the NLRP3 inflammasome.^39^ Studies have demonstrated that the UAF1 deubiquitinase complex removes K48‐linked ubiquitin chains from NLRP3, thereby inhibiting ubiquitin‐mediated degradation and promoting the activation of the NLRP3 inflammasome.^40^ Additionally, OTUD6A interacts with the NACHT domain of NLRP3, facilitating the removal of K48‐linked polyubiquitin chains at residues K430 and K689, which enhances NLRP3 stability in macrophages.^41^ Conversely, deubiquitination modifications also play a pivotal role in suppressing NLRP3 inflammasome activation. Research has indicated that USP19 and USP22 both prevent the activation of NLRP3 by cleaving its polyubiquitin chains.^42^, ^43^ Furthermore, STAMBP (STAM‐binding protein) contributes to this regulation process by deubiquitylating NLRP3 and suppressing its expression levels, thus inhibiting NLRP3 inflammasome activation.^44^ In the present study, NLRP3 was found to be deubiquitinated by USP20 in cardiomyocyte induced by LPS/Nig. USP20‐mediated deubiquitination of NLRP3 prevents its interaction with ASC, thereby blocking the activation of inflammasome and subsequent pyroptosis. We hypothesize that the removal of K63‐linked ubiquitin chains at residue K243 may induce a conformational change in NLRP3, hindering its effective binding to ASC. Overall, our research expands current understanding regarding the regulation of NLRP3 through deubiquitination processes.

Given the role of USP20 in inhibiting NLRP3 activation, targeting USP20 may be a potential therapeutic strategy for septic cardiomyopathy. However, no USP20 specific agonists are available in preclinical studies now. Currently, AAV9 is widely used in the field of gene therapy. By loading therapeutic genes into AAV9, it can be delivered to target cells to treat various genetic disorders, cancer, and other conditions.^45^, ^46^, ^47^, ^48^ AAV9 gene therapy has also achieved certain results in cardiovascular research.^49^, ^50^ We have demonstrated that overexpression of USP20 in cardiomyocyte via AAV9 injection does not result in any significant impairment of liver function, kidney function, or lipid metabolism and exhibits significant therapeutic effects in the treatment of septic cardiomyopathy, which provides a potential breakthrough for the management of septic cardiomyopathy. However, AAV9 gene therapy also encounters various challenges and limitations that need to be addressed. The pathological environment of sepsis is highly complex, involving excessive systemic inflammatory response and endothelial barrier dysfunction. These pathophysiological changes may compromise the transduction efficiency of AAV9 or affect the expression of the therapeutic gene within the target tissue.

In conclusion, our study has demonstrated that cardiomyocyte‐specific USP20 deubiquitylates NLRP3 by effectively removing K63‐linked ubiquitin chains from NLRP3 within cardiomyocytes. This mechanism inhibits the interaction between NLRP3 and ASC, consequently suppressing NLRP3 activation and subsequent pyroptosis, which ultimately contributes to the mitigation of septic cardiomyopathy. USP20 may represent a novel therapeutic target for the treatment of septic cardiomyopathy.

## AUTHOR CONTRIBUTIONS

Weijian Huang, Xueli Cai and Xiangtao Zheng contributed to the literature search and study design. Shanshan Dai, Yucong Zhang, Ziyi Huang, Yunxuan Chen, Zexin Yang, Ruihan Zheng, Keke Ye and Lingfeng Zhong performed the experiments and analysed the data. Xueli Cai and Xiangtao Zheng provided technical help. Shanshan Dai participated in the drafting of the article. All authors agree to be accountable for all aspects of work ensuring integrity and accuracy.

## CONFLICT OF INTEREST STATEMENT

The authors declare no conflicts of interest.

## ETHICAL APPROVAL

All experimental procedures received approval from the Laboratory Animal Ethics Committee and the Laboratory Animal Centre of the First Affiliated Hospital of Wenzhou Medical University (WYYY‐IACUC‐AEC‐2024‐106).

## Supporting information



Online Supplementary Table S1‐S5

Online Supplementary Figure S1‐S8

## Data Availability

All data needed to evaluate the conclusions in this study are presented in this manuscript or the supplementary information. The materials described in this study are either commercially available or available upon reasonable request from the corresponding authors.
