# Supplementary material for: Cardiomyocyte USP20 alleviates septic cardiomyopathy by deubiquitinating and inhibiting NLRP3 activity
Source: Clin Transl Med. 2025 Oct 3;15(10):e70494. doi: 10.1002/ctm2.70494 (PMC12493026; doi:10.1002/ctm2.70494)
Supplement: Supplementary file 1 — Online Supplementary Table S1‐S5 [file CTM2-15-e70494-s002.docx]

**Cardiomyocyte USP20 alleviates septic cardiomyopathy by deubiquitinating and inhibiting NLRP3 activity**

***Supplementary Information***

Supplementary Information Contents:

Supplementary Table S1-S5

Supplementary Figure S1-S8

**Supplementary Table S1**

Primer sequences used for real-time qPCR analysis

| Gene | Species | Sequence (Forward) | Sequence (Reverse) |
| --- | --- | --- | --- |
| *USP20* | Mouse | GCCTTCATCGTGGAGTACATCAGAC | CAGCAGCAAAGAAAGCAGCAAGG |
| *Actinb* | Mouse | CCGTGAAAAGATGACCCAGA | TACGACCAGAGGCATACAG |

**Supplementary Table S2**

Echocardiographic parameters in LPS-challenged mouse experiment

|  | | Saline | | | LPS | | | |  |  |
| --- | --- | --- | --- | --- | --- | --- | --- | --- | --- | --- |
|  | | USP20^fl/fl^ | | USP20CKO | USP20^fl/fl^ | | USP20CKO | |  |  |
|  | | n=6 | | n=6 | n=6 | | n=6 | |  |  |
| Heart Rate  Ejection Fraction  Fractional Shortening  LVID;d, (mm) | | 470.54±38.18  67.18±5.20  36.44±4.03  3.39±0.49 | | 467.25±33.09 ^ns^  67.12±4.73 ^ns^  36.33±3.65 ^ns^  3.28±0.23^ns^ | | | 376.83±45.19**  51.07±2.88***  25.25±1.71***  3.29±0.31^ns^ | | 350.27±38.02 ^NS^  33.37±6.14^###^  15.50±3.06^###^  3.73±0.36^NS^ | |
| LVID;s, (mm) | | 2.16±0.40 | | 2.09±0.20^ns^ | | | 2.46±0.25^ns^ | | 3.15±0.40^##^ | |
| LVAW;d, (mm) | | 0.76±0.10 | | 0.76±0.08^ns^ | | | 0.82±0.15^ns^ | | 0.70±0.11^NS^ | |
| LVAW;s, (mm) | | 1.16±0.14 | | 1.13±0.11^ns^ | | | 1.14±0.10^ns^ | | 0.93±0.14^#^ | |
| IVS;d, (mm) | | 0.78±0.11 | | 0.84±0.07^ns^ | | | 0.97±0.32^ns^ | | 0.87±0.21^NS^ | |
| IVS;s, (mm) | | 1.13±0.17 | | 1.19±0.09^ns^ | | | 1.36±0.23^ns^ | | 0.93±0.11^###^ | |
| CO, (mL/min) | | 16.19±1.62 | | 15.23±1.63^ns^ | | | 9.36±1.07*** | | 6.77±1.02^#^ | |

LPS, lipopolysaccharide; LVID, left ventricular internal diameter; LVAW, left ventricular anterior wall; IVS, interventricular septum; CO, cardiac output; d, diastole; s, systole. ns, represents p>0.05 vs USP20^fl/fl^ + Saline; ***, p<0.001 vs USP20^fl/fl^ + Saline; NS, represents p>0.05 vs USP20^fl/fl^ + LPS; #, p<0.05 vs USP20^fl/fl^ + LPS; ##, p<0.01 vs USP20^fl/fl^ + LPS; ###, p<0.001 vs USP20^fl/fl^ + LPS.

**Supplementary Table S3**

Echocardiographic parameters in CLP-challenged mouse experiment

|  | Sham | | CLP | |
| --- | --- | --- | --- | --- |
|  | USP20^fl/fl^ | USP20CKO | USP20^fl/fl^ | USP20CKO |
|  | n=6 | n=6 | n=6 | n=6 |
| Heart Rate  Ejection Fraction  Fractional Shortening  LVID;d, (mm) | 466.04±52.04  61.51±6.01  32.31±4.42  3.42±0.28 | 467.47±49.68 ^ns^  60.84±6.48 ^ns^  31.58±4.40 ^ns^  3.11±0.28^ns^ | 363.43±41.73**  50.92±4.24*  25.18±2.72*  3.22±0.39^ns^ | 372.25±55.49 ^NS^  39.78±4.33^##^  18.80±2.40^#^  3.37±0.30^NS^ |
| LVID;s, (mm) | 2.28±0.21 | 2.07±0.24^ns^ | 2.41±0.30^ns^ | 2.74±0.25^NS^ |
| LVAW;d, (mm) | 0.84±0.15 | 0.92±0.16^ns^ | 0.92±0.24^ns^ | 0.69±0.10^NS^ |
| LVAW;s, (mm) | 1.28±0.15 | 1.28±0.17^ns^ | 1.26±0.27^ns^ | 0.90±0.11^#^ |
| IVS;d, (mm) | 0.84±0.14 | 0.81±0.09^ns^ | 0.78±0.23^ns^ | 0.83±0.19^NS^ |
| IVS;s, (mm) | 1.13±0.10 | 1.09±0.08^ns^ | 0.90±0.10*** | 0.74±0.05^#^ |
| CO, (mL/min) | 12.42±1.87 | 12.94±2.47^ns^ | 8.62±2.11* | 5.11±1.39^#^ |

CLP, caecal ligation and puncture; LVID, left ventricular internal diameter; LVAW, left ventricular anterior wall; IVS, interventricular septum; CO, cardiac output; d, diastole; s, systole. ns, represents p>0.05 vs USP20^fl/fl^ + Sham; *, p<0.05 vs USP20^fl/fl^ + Sham; ***, p<0.001 vs USP20^fl/fl^ + Sham; NS, represents p>0.05 vs USP20^fl/fl^ + CLP; #, p<0.05 vs USP20^fl/fl^ + CLP;

**Supplementary Table S4**

Echocardiographic parameters in LPS-challenged mice with treatment of AAV9 that specifically overexpress the target gene in cardiomyocytes (Empty Vector, cTnT-USP20)

|  | Saline | LPS | |
| --- | --- | --- | --- |
|  | WT | WT | WT |
|  | AAV9-cTnT-Flag-EV | AAV9-cTnT-Flag-EV | AAV9-cTnT-FlagUSP20 |
|  | n=6 | n=6 | n=6 |
| Heart Rate  Ejection Fraction  Fractional Shortening  Diameter;d, (mm) | 501.85±17.96  72.21±7.07  40.46±5.91  3.08±0.41 | 420.94±58.16**  48.92±3.91***  24.08±2.24***  3.50±0.27^ns^ | 486.49±13.22^#^  63.13±1.94^###^  33.29±1.42^##^  3.31±0.20^NS^ |
| Diameter;s, (mm) | 1.84±0.30 | 2.66±0.29*** | 2.21±0.13^#^ |
| LVAW;d, (mm) | 0.78±0.13 | 0.73±0.10^ns^ | 0.87±0.10^NS^ |
| LVAW;s, (mm) | 1.23±0.27 | 1.09±0.10^ns^ | 1.31±0.12^NS^ |
| IVS;d, (mm) | 0.80±0.26 | 0.76±0.12^ns^ | 0.79±0.17^NS^ |
| IVS;s, (mm) | 1.16±0.29 | 0.92±0.15^ns^ | 1.10±0.11^NS^ |
| CO, (mL/min) | 14.28±3.10 | 9.58±0.80** | 12.80±1.05^#^ |

LPS, lipopolysaccharide; EV, Empty Vector; LVID, left ventricular internal diameter; LVAW, left ventricular anterior wall; IVS, interventricular septum; CO, cardiac output; d, diastole; s, systole. ns, represents p>0.05 vs WT + AAV9-cTnT-Flag-EV + Saline; *, p<0.05 vs WT + AAV9-cTnT-Flag-EV + Saline; **, p<0.01 vs WT + AAV9-cTnT-Flag-EV + Saline; ***, p<0.001 vs WT + AAV9-cTnT-Flag-EV + Saline; ****, p<0.0001 vs WT + AAV9-cTnT-Flag-EV + Saline; NS, represents p>0.05 vs WT + AAV9-cTnT-Flag-EV + LPS; #, p<0.05 vs WT + AAV9-cTnT-Flag-EV + LPS;##, p<0.01 vs WT + AAV9-cTnT-Flag-EV + LPS; ###, p<0.001 vs WT + AAV9-cTnT-Flag-EV + LPS; ####, p<0.0001 vs WT + AAV9-cTnT-Flag-EV + LPS.

**Supplementary Table S5**

Echocardiographic parameters in LPS-challenged NLRP3^-/-^ mouse experiment with treatment of AAV9-cTnT-Flag-USP20^oe^

|  | LPS | |
| --- | --- | --- |
|  | NLRP3^-/-^ | NLRP3^-/-^ |
|  | AAV9-cTnT-Flag-EV | AAV9-cTnT-Flag-USP20^oe^ |
|  | n=6 | n=6 |
| Heart Rate  Ejection Fraction  Fractional Shortening  Diameter;d, (mm) | 370.47±55.35  50.46±7.83  25.35±4.64  3.84±0.47 | 396.80±56.48 ^NS^  52.86±4.22 ^NS^  26.55±2.73 ^NS^  3.52±0.37^NS^ |
| Diameter;s, (mm) | 2.87±0.47 | 2.58±0.26^NS^ |
| LVAW;d, (mm) | 0.78±0.14 | 0.13±0.08^NS^ |
| LVAW;s, (mm) | 0.60±0.08 | 0.70±0.06* |
| IVS;d, (mm) | 0.87±0.12 | 0.88±0.08^NS^ |
| IVS;s, (mm) | 0.67±0.12 | 0.73±0.07^NS^ |
| CO, (mL/min) | 11.65±2.65 | 12.33±3.02^NS^ |

LPS, lipopolysaccharide; LVID, left ventricular internal diameter; LVAW, left ventricular anterior wall; IVS, interventricular septum; CO, cardiac output; d, diastole; s, systole. NS, represents p>0.05 vs NLRP3^-/-^ + AAV9-cTnT-Flag-EV + LPS; *, p<0.05 vs NLRP3^-/-^ + AAV9-cTnT-Flag-EV + LPS.

**Supplementary Table S6**

| Protein names | Gene names | Unique peptides | Sequence coverage [%] | Mol. weight [kDa] | Score |
| --- | --- | --- | --- | --- | --- |
| NACHT, LRR and PYD domains-containing protein 3 | Nlrp3 | 17 | 12.3 | 118.27 | 154.04 |
| Ubiquitin carboxyl-terminal hydrolase 20 | Usp20 | 33 | 33.4 | 102.14 | 298.75 |


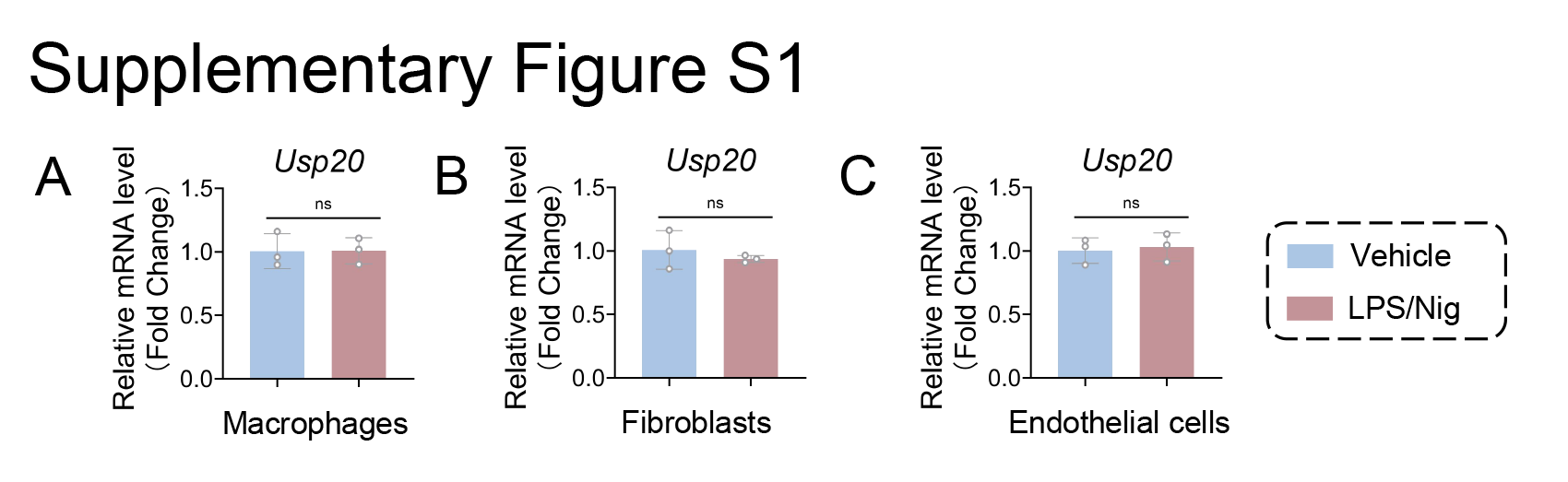


**Supplementary Figure S1**

**(A)** The mRNA expression levels of Usp20 in macrophages induced by LPS/Nig were respectively assessed by PCR. n = 3.

**(B)** The mRNA expression levels of Usp20 in fibroblasts induced by LPS/Nig were respectively assessed by PCR. n = 3.

**(C)** The mRNA expression levels of Usp20 in endothelial cells induced by LPS/Nig were respectively assessed by PCR. n = 3.

Data are expressed as the mean ± SD. *P* > 0.05, ns: no differences.


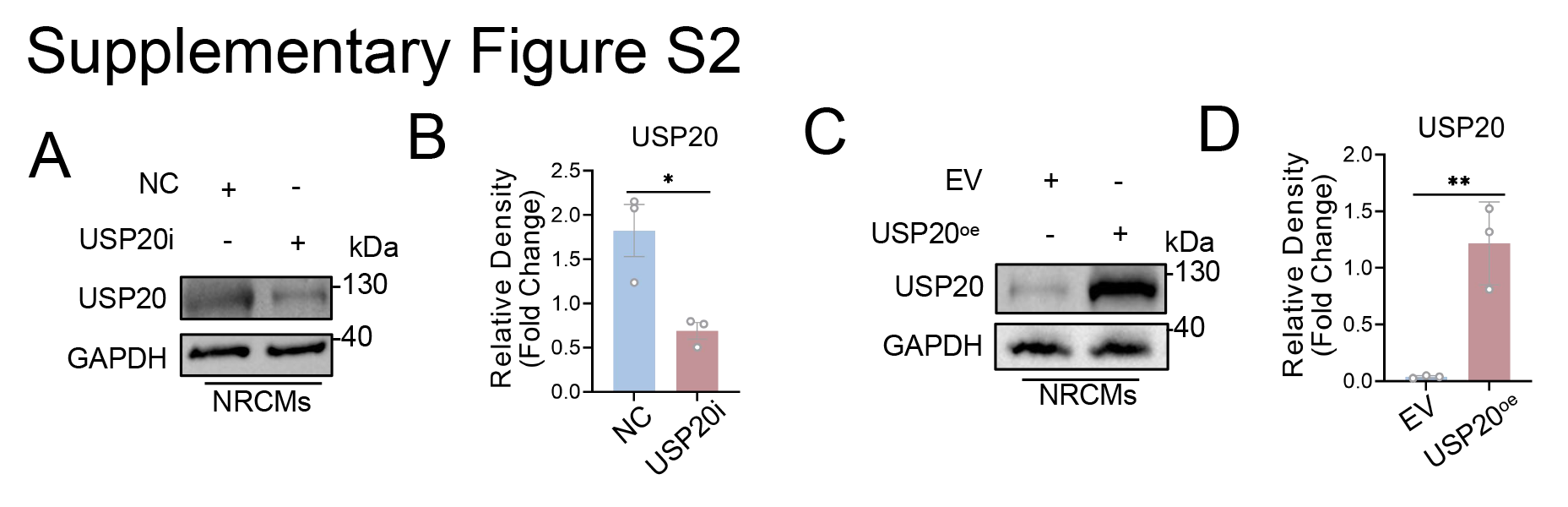


**Supplementary Figure S2**

**(A-B)** The protein expression level of USP20 in NRCMs overexpressing USP20 by utilizing USP20 plasmid (A) and the statistical results (B). n = 3.

**(C-D)** The protein expression level of USP20 in NRCMs following the silencing of USP20 utilizing siRNA (C) and the statistical results (D). n = 3.

Data are expressed as the mean ± SD. **, *P* < 0.01; *, *P* < 0.05.


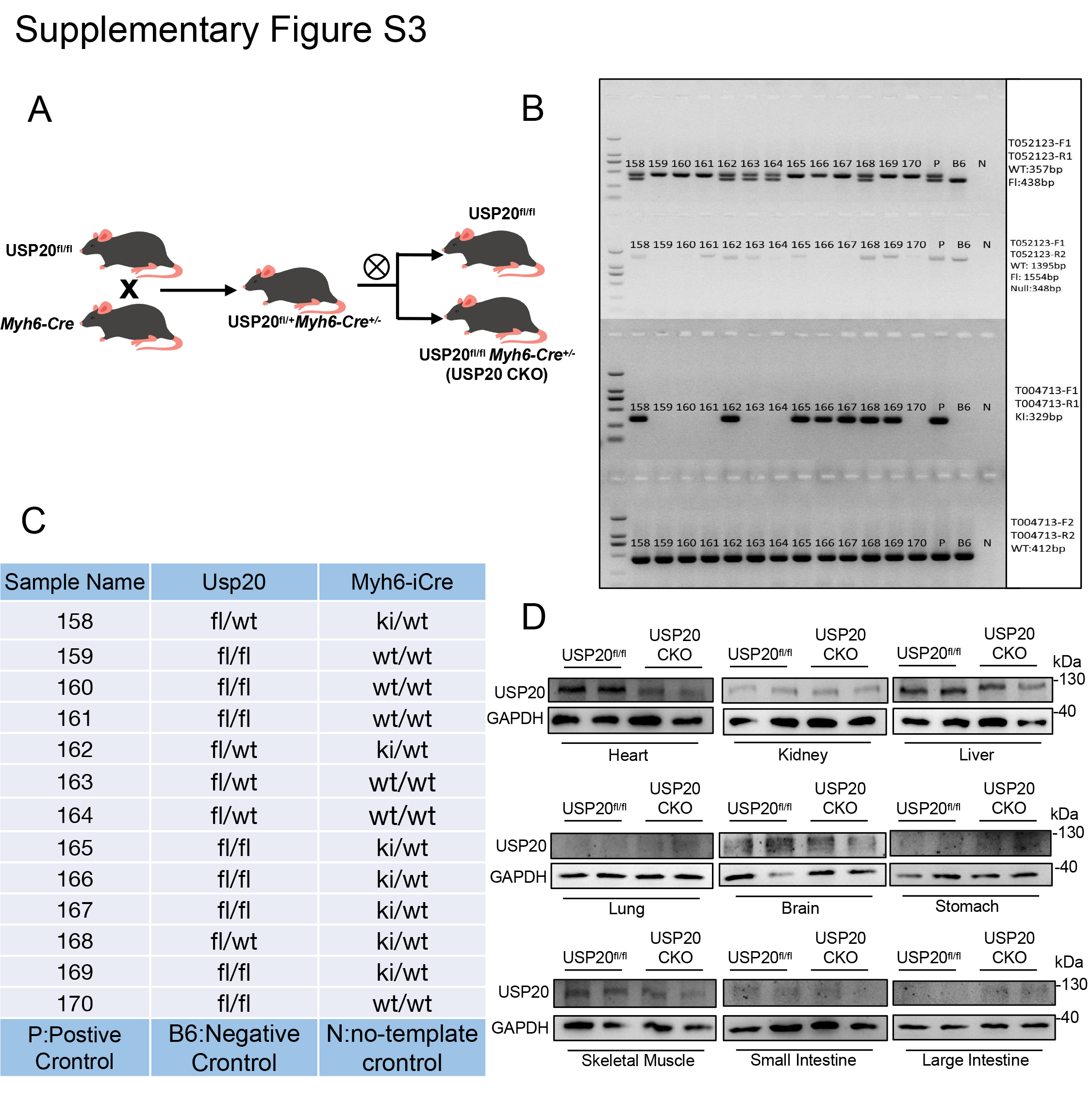


**Supplementary Figure S3**

**(A)** Schematic diagram of USP20 cardiomyocyte specific knockout (USP20CKO) mice construct.

(**B**) The primers of Usp20 (WT:357bp, FI:438bp) and Myh6-Cre (WT:412bp, KI:329bp) were respectively used for PCR to identify the genotype of mice. (P: Positive control; B6: Negative control; N: No-template control).

(**C**) Table of USP20CKO mouse identification results.

(**D**) Representative western blotting of USP20 in heart, kidney, liver, lung, brain, stomach, skeletal muscle, small intestine and lagre intestine tissues of USP20^fl/fl^ and USP20CKO mice.

**
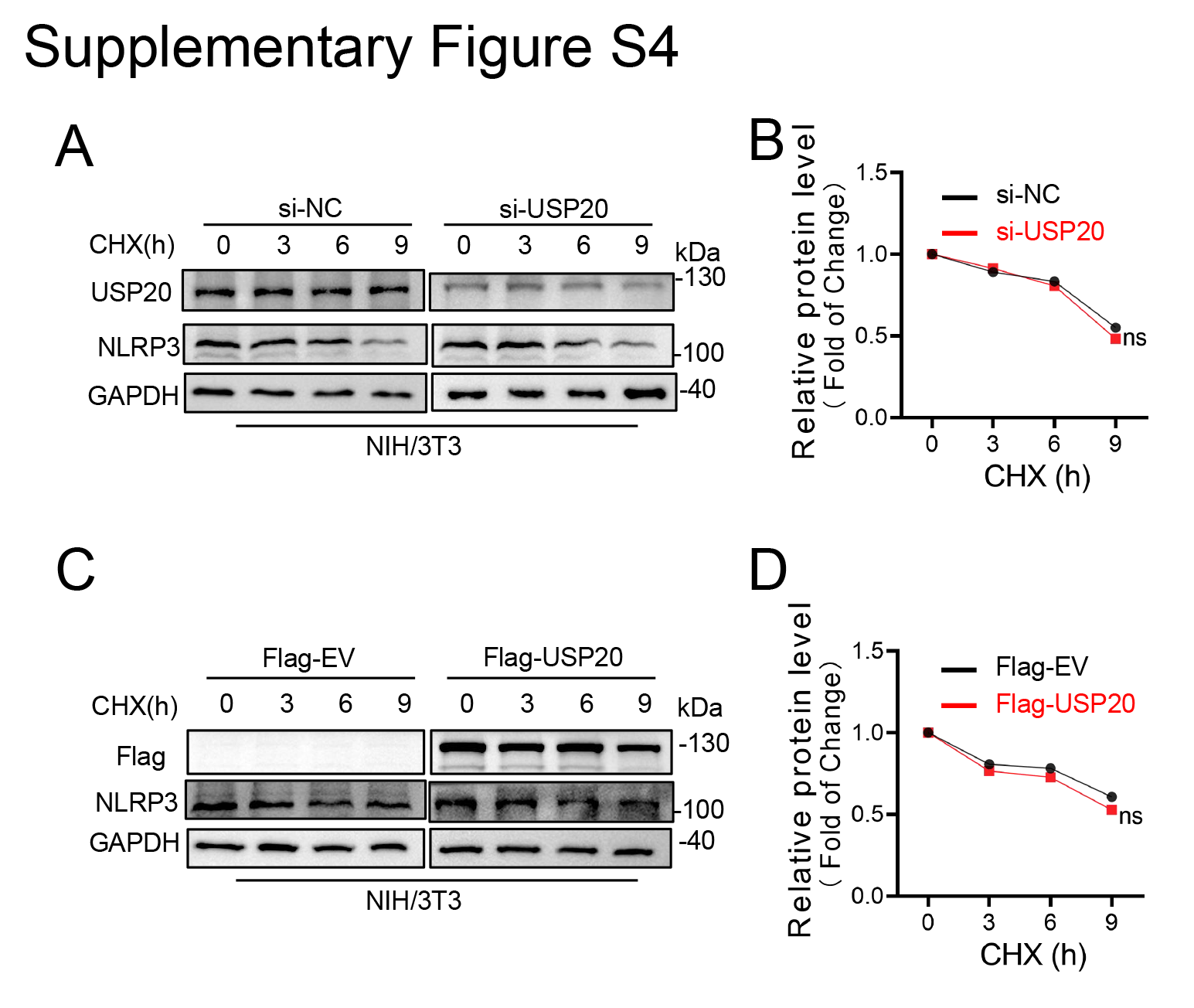
**

**Supplementary Figure S4**

**(A-B)** Protein expression level of USP20 and NLRP3 in NIH/3T3 cells transfected with si-USP20 or si-NC for different time **(**A**)** and statistical results **(**B**).** n = 3.

**(C-D)** Protein expression level of USP20 and NLRP3 protein in NIH/3T3 cells transfected with Flag-USP20 plasmids or Flag-EV plasmids for different time **(C)** and statistical results **(D).** n = 3.

Data are expressed as the mean ± SD. *P* > 0.05, ns: no differences.

**
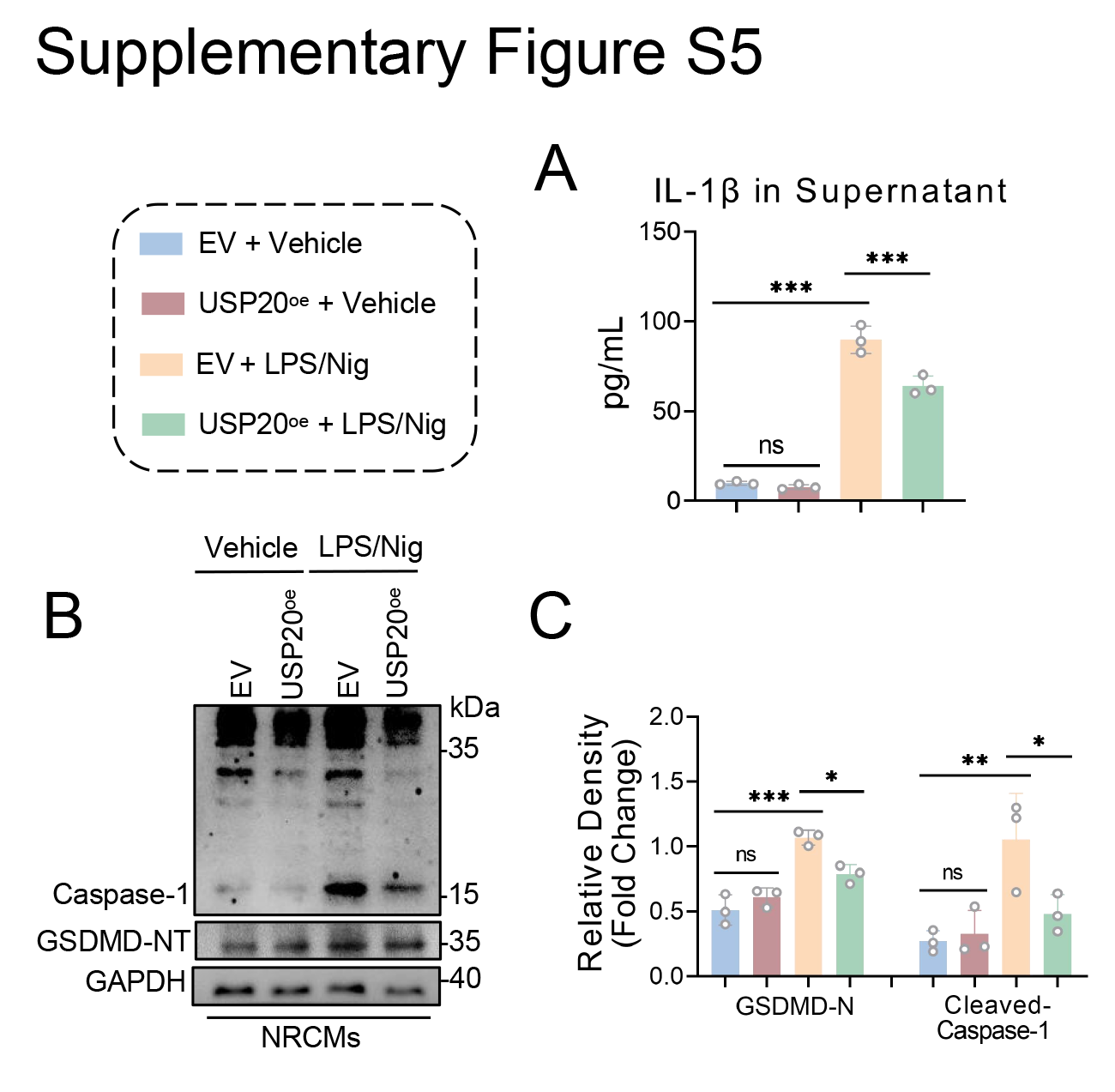
**

**Supplementary Figure S5**

After transfection with an encoding USP20 plasmid (USP20^oe^) or a control empty vector (EV) to overexpress USP20, cells were treated with LPS/Nig.

**(A)** IL-1β level in supernatants of each group. n = 3.

**(B-C)** Protein expressions level of Caspase-1 and GSDMD-NT in NRCMs **(**B**)** and statistical results **(**C**).** n = 3.

Data are expressed as the mean ± SD. ***, *P* < 0.001; **, *P* < 0.01; *, *P* < 0.05, *P* > 0.05, ns: no differences.

**
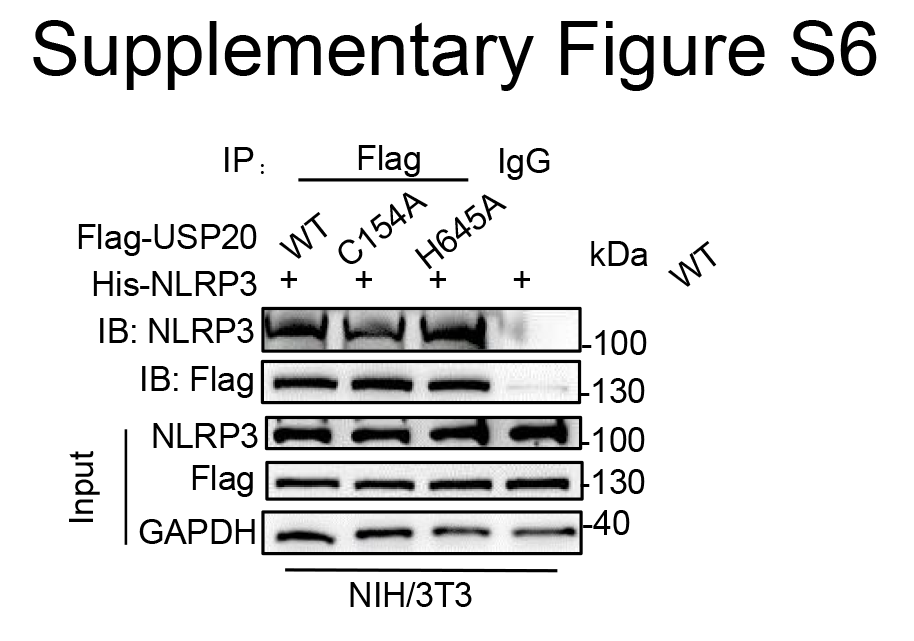
**

**Supplementary Figure S6**

Immunoprecipitation of NLRP3 in NIH/3T3 cells that co-transfected with plasmids encoding His-NLRP3, USP20-WT, USP20-C154A and USP20-H645A.


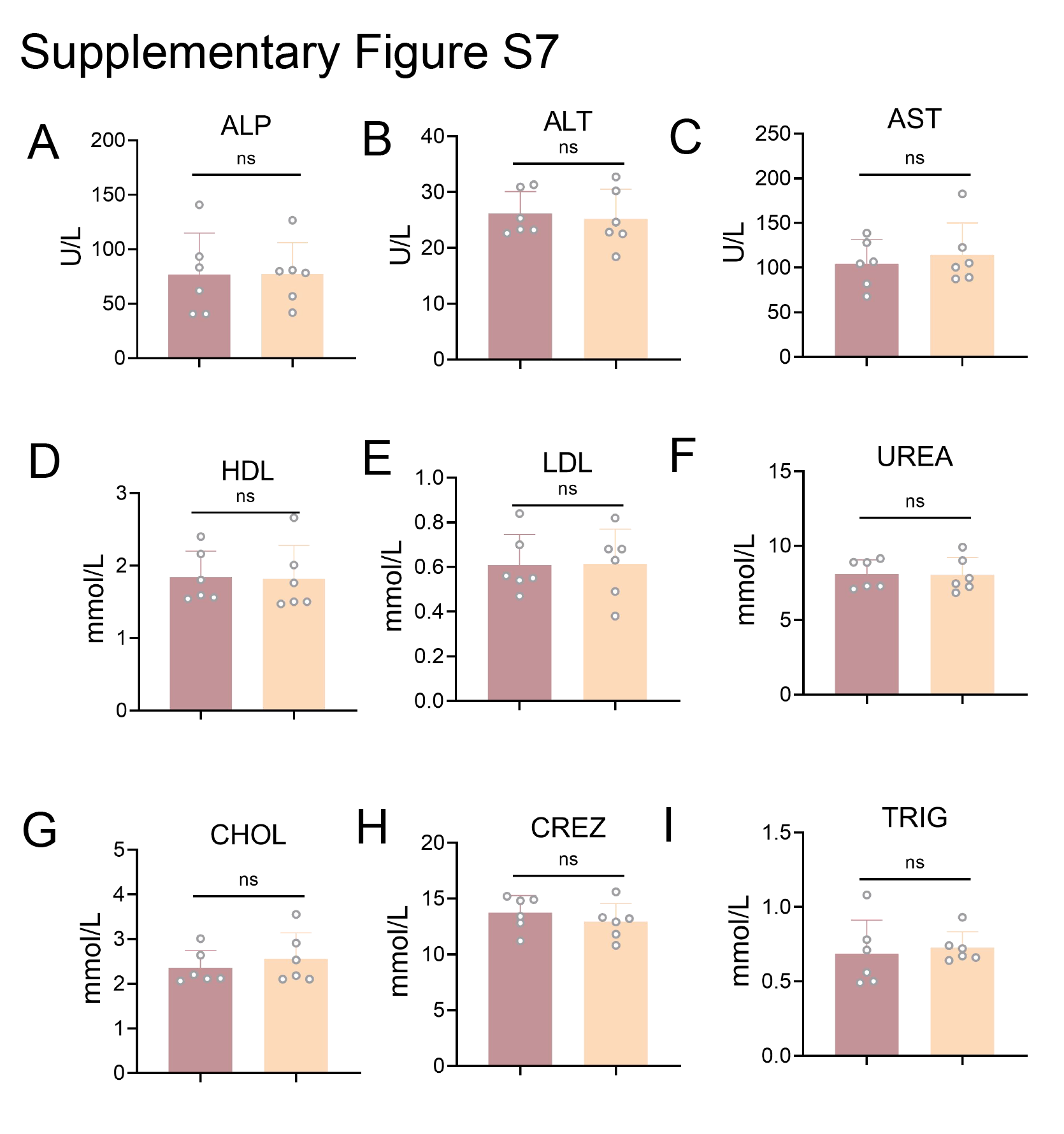


**Supplementary Figure S7**

The levels of ALP, ALT, AST, HDL, LDL, UREA ,CHOL, CREZ and TRIG in serum from mice with or without cardiac-specific overexpression of USP20. n = 6.


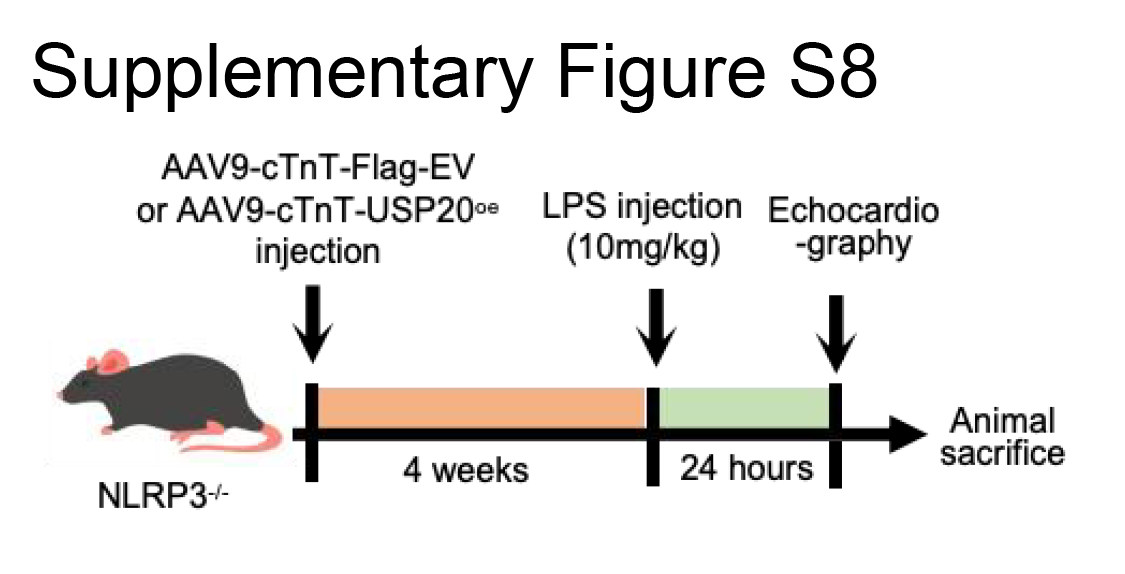


**Supplementary Figure S8**

Schematic diagram of LPS-induce septic myocardial injury mouse model in NLRP3 knockout (NLRP3^-/-^) mice overexpressing USP20 in cardiomyocytes.
